# APOBEC3A is a prominent cytidine deaminase in breast cancer

**DOI:** 10.1371/journal.pgen.1008545

**Published:** 2019-12-16

**Authors:** Luis M. Cortez, Amber L. Brown, Madeline A. Dennis, Christopher D. Collins, Alexander J. Brown, Debra Mitchell, Tony M. Mertz, Steven A. Roberts

**Affiliations:** 1 School of Molecular Biosciences and Center for Reproductive Biology, Washington State University, Pullman, WA, United States of America; 2 Department of Biochemistry and Molecular Biology, University of Kansas Medical Center, Kansas City, KS, United States of America; 3 School of Molecular Biosciences, Washington State University-Vancouver, Vancouver, WA, United States of America; The Children's Hospital of Philadelphia, UNITED STATES

## Abstract

APOBEC cytidine deaminases are the second-most prominent source of mutagenesis in sequenced tumors. Previous studies have proposed that APOBEC3B (A3B) is the major source of mutagenesis in breast cancer (BRCA). We show that APOBEC3A (A3A) is the only APOBEC whose expression correlates with APOBEC-induced mutation load and that A3A expression is responsible for cytidine deamination in multiple BRCA cell lines. Comparative analysis of A3A and A3B expression by qRT-PCR, RSEM-normalized RNA-seq, and unambiguous RNA-seq validated the use of RNA-seq to measure APOBEC expression, which indicates that A3A is the primary correlate with APOBEC-mutation load in primary BRCA tumors. We also demonstrate that A3A has >100-fold more cytidine deamination activity than A3B in the presence of cellular RNA, likely explaining why higher levels of A3B expression contributes less to mutagenesis in BRCA. Our findings identify A3A as a major source of cytidine deaminase activity in breast cancer cells and possibly a prominent contributor to the APOBEC mutation signature.

## Author summary

APOBEC-signature mutations are over-represented in ~15% of all sequenced tumors and in many tumors constitute more than 50% of all mutations. Furthermore, APOBEC activity likely contributes to the occurrence of primary driver and late sub-clonal driver mutations, and mutations that contribute to metastasis and resistance to chemotherapeutics in cancer. To clarify the roles of individual APOBEC family members to cancer mutagenesis, we compared the expression of each APOBEC3 gene in a panel of breast cancer cell lines to the corresponding amount of APOBEC-induced mutation, finding that only A3A positively correlated. We found that A3A provides a significant amount of the cell’s total cytidine deamination activity in breast cancer cell lines. We also show that unlike other APOBECs, A3A is not inhibited by RNA, which partly explains its disproportionate contribution to cellular APOBEC activity. Further bioinformatics analysis determined that A3A expression is a major correlate with APOBEC-induced mutagenesis in primary tumors from multiple cancer types. This establishes A3A as a likely contributor to cancer mutagenesis.

## Introduction

Apolipoprotein B mRNA-editing enzyme catalytic polypeptide-like (APOBEC) cytidine deaminases are a major cause of mutation in approximately 15% of sequenced human tumors [1–3]. While these enzymes play diverse physiological roles, including contributing to antibody diversification and restricting viral pathogenesis [4], their off-target activity towards genomic DNA frequently causes oncogenic *PIK3CA* mutations [5], creates genetic diversity within cancer genomes [6], and contributes to chemotherapeutic resistance [7]. In addition, the high frequency of APOBEC-induced genome instability in tumors has led to exploration of APOBEC-activity as a potential target for cancer therapeutics[8–10]. APOBECs induce mutations in a sequence-specific manner, with the majority of APOBEC3 family members preferentially deaminating the central cytidine in TCW trinucleotide motifs (where W = A or T; deaminated based is underlined) within single stranded (ss) DNA [4]. Consequently, APOBEC-mutated tumors are largely identified by a dramatic over-representation of C to T and C to G substitutions within TCW sequences [11, 12], which forms the basis of APOBEC-induced mutation signatures 2 and 13 in cancer genomes [3]. APOBEC-induced mutations in tumors display replication strand bias [13–15], high density clustering around DNA translocation sites (termed *kataegis*) [11, 12], and frequently occur in hairpin forming sequences [16], all of which have been recapitulated in experimental systems exogenously expressing APOBECs [8, 17–21], which further reinforces the consensus that APOBECs are the underlying cause of this distinctive class of tumor-specific genetic instability.

Identifying the specific cytidine deaminases responsible for cancer mutagenesis and the relative contribution of each APOBEC to genomic instability in different cancer types is an ongoing area of research with many clinical implications. However, significant homology and functional redundancy between the 11 APOBEC enzymes encoded by the human genome has hindered such efforts. Nine APOBECs display catalytic activity towards ssDNA and only APOBEC3G (A3G) and Activation-Induced Cytidine Deaminase (AID) deaminate cytidines in sequences differing from the canonical TCW motif that is deaminated by the remaining seven enzymes [4]. Recent efforts to characterize the potential mutagenic activity of APOBECs have focused primarily on cellular localization and APOBEC mRNA expression levels [22, 23]. Of the APOBECs capable of causing APOBEC-signature mutations, APOBEC3DE (A3DE) and APOBEC3F (A3F) are predominantly cytoplasmic, A3A and APOBEC3C (A3C) display pan-cellular localization, and only APOBEC1, A3B and APOBEC3H (A3H) haplotype I are primarily nuclear [24–26]. A3B is also highly expressed in BRCA and reduction of A3B expression by short hairpin RNA (shRNA) decreased cytidine deaminase activity and levels of deoxyurdine (dU) in genomic DNA in some BRCA cell lines [22]. In addition, a positive correlation between A3B expression and the total mutation load in APOBEC-mutated tumor types indicates A3B may be the predominant source of APOBEC-induced mutations in multiple cancer types including breast, bladder, cervical, lung (adeno- and squamous cell), and head-and-neck cancers [2, 22].

Approximately 22% of humans carry a deletion polymorphism resulting in loss of the *APOBEC3B* gene [27]. Sequencing of BRCA tumors from individuals with the *APOBEC3B* deletion polymorphism revealed that these tumors frequently contained APOBEC-induced mutations and often at higher abundance than tumors from non-carriers [28]. Additionally, deletion of exon 5 of *APOBEC3B* is common in human BRCA [29], which results in ablation of A3B protein. Thus, additional APOBEC enzymes besides A3B almost certainly cause APOBEC-signature mutations during tumor development. Bioinformatics analysis of APOBEC-induced mutations from a limited number of A3B deleted BRCA tumors revealed that the only tumors displaying the APOBEC mutation signature also contained the nuclear A3H haplotype I [23], providing correlative evidence that this protein may be the additional source of mutagenesis. The protein abundance of A3H haplotype I, however, is low due to impaired stability of the protein [30]. Alternatively, endogenous A3A was previously shown to be capable of deaminating genomic DNA in human macrophages and myeloid cells [31, 32]. In addition, A3A and A3B prefer tetranucleotide motifs, YTCA and RTCA (Y = T or C and R = G or A) respectively, when inducing mutations in model systems. However, over-representation of mutations in the YTCA motif predominates in a variety of cancers [33] as well as among mutations actively acquired in BRCA cell lines [34], suggesting A3A may likewise contribute to cancer mutagenesis. Still, this association currently remains correlative. Ultimately, multiple lines of conflicting data have led to an unclear understanding as to the relative contributions of individual APOBECs to mutagenesis in cancer.

Here, we provide evidence that indicates A3A may be a major cause of APOBEC-induced mutation in BRCA. Using analysis of mutations from 28 whole exome sequenced BRCA cell lines and the corresponding expression of APOBEC3 family members determined by qRT-PCR, we show that only A3A expression was elevated in APOBEC-mutated lines compared to non-APOBEC-mutated lines and that the overall abundance of APOBEC-induced mutations linearly correlates with A3A expression. We also found that an A3B-targetting shRNA, which was used throughout the seminal study showing that A3B activity is responsible for cytidine deamination activity, accumulation of dU, and C-to-T mutations in BRCA [22], also decreases endogenous A3A mRNA levels up to 13.8-fold. These finding prompted us to reevaluate the relative contribution of APOBECs to BRCA mutagenesis. By selective depletion of A3A and A3B from cell populations with shRNAs, we demonstrate that A3A is responsible for the majority of cytidine deaminase activity in extracts from multiple BRCA cell lines, despite A3B’s higher expression. We provide evidence this discordance between expression and deaminase activity results from higher intrinsic activity of A3A and severe inhibition of A3B by cellular RNA. Finally, we used a novel analysis of RNA-seq reads limited to the unique 5’ and 3’ portions of the A3A and A3B transcripts (termed unambiguous RNA-seq) to validate that RNA-seq provides an accurate assessment of APOBEC expression, which subsequently reveals a greater positive correlation between A3A expression and APOBEC-signature mutations in primary BRCA tumors sequenced by The Cancer Genome Atlas (TCGA) than exists between A3B expression and mutagenesis. These results indicate that A3A expression is likely a principal determinant of the APOBEC mutation signature in BRCA.

## Results

### APOBEC3A is a probable source of APOBEC-signature mutations in APOBEC3B null cell lines

**Fig 1:**
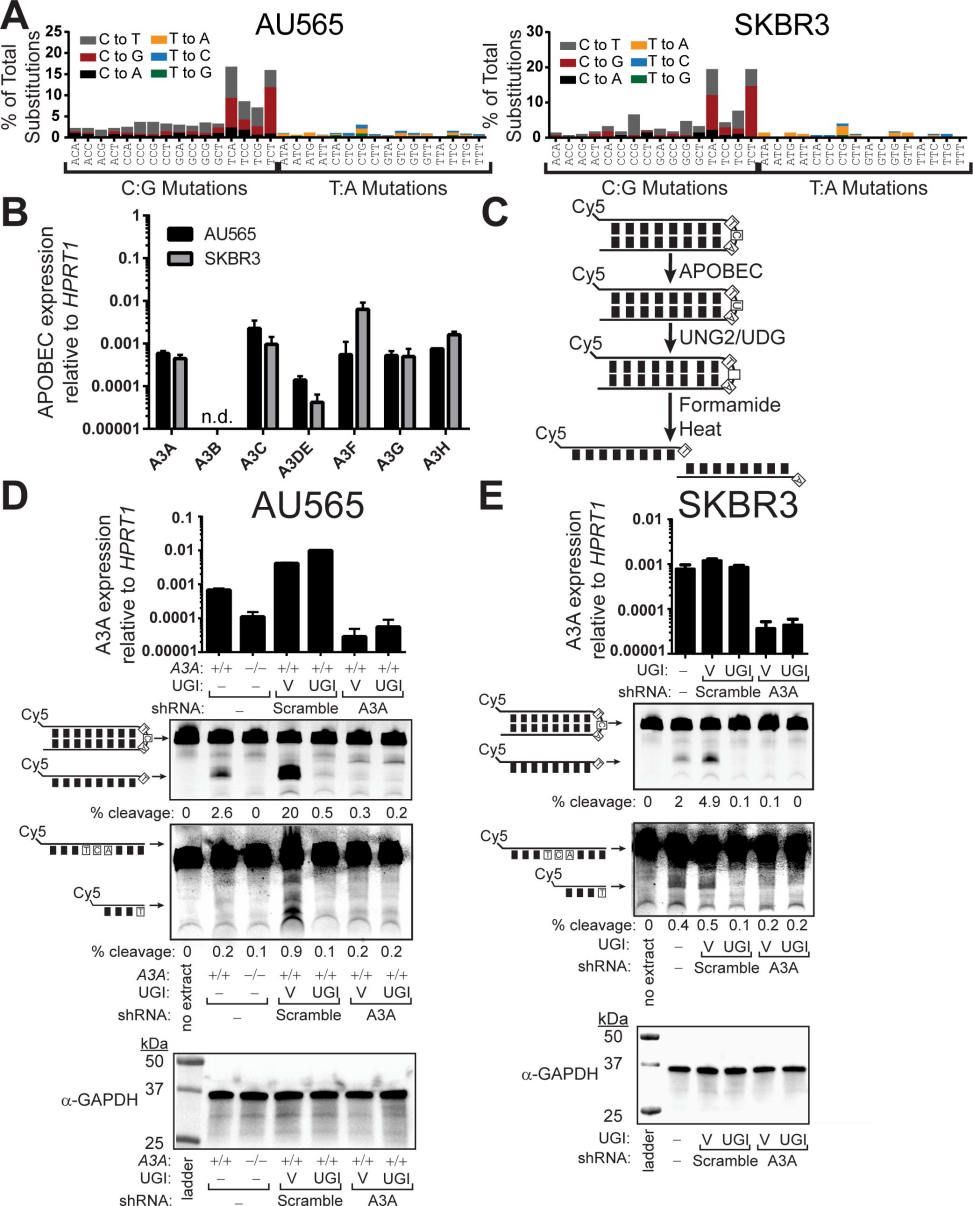
APOBEC3A is the predominant cytidine deaminase in BRCA cell lines lacking APOBEC3B. (A) The mutation profile of AU565 and SKBR3 BRCA cell lines. (B) mRNA expression level of individual APOBEC3 family members relative to *HPRT1* expression in AU565 (black) and SKBR3 (grey). Bars indicate the mean values of 3 replicate measurements. Error bars indicate the standard error of the mean (SEM) of these measurements. n.d. indicates “not detected.” Similar results were obtained using *TBP* instead of *HPRT1* as the internal reference gene (S2 Table). (C) Schematic of *in vitro* cytidine deaminase assay. (D) AU565, AU565 cells containing a CRISPR-Cas9 mediated disruption of *APOBEC3A* (-/-), and (E) SKBR3 BRCA cell lines either un-transduced or expressing scramble control, A3A shRNA, or A3B shRNA were tested for cytidine deaminase activity on a hairpin or linear substrate containing a YTCA APOBEC target motif. Each cell line was additionally transduced to express a vector control or uracil glycosylase inhibitor (UGI) as indicated. 40 μg of total protein was incubated with 0.25 μM of hairpin substrate for 24 hrs at 37°C, prior to heating the samples at 95°C for 10 min and separating substrate from cleavage product on a denaturing polyacrylamide gel. Knockdown specificity was confirmed by qRT-PCR and equal protein amount in each reaction was confirmed via α-GAPDH western.

To identify APOBECs besides A3B that produce mutagenesis in BRCA, we evaluated the mutation signatures of a panel of BRCA cell lines to identify A3B null lines that are APOBEC mutated. We found that autologous BRCA cell lines SKBR3 and AU565 display a strong APOBEC mutation signature, as indicated by a high proportion of mutations involving C to T and C to G substitutions in TCW trinucleotide sequences (Fig 1A and S1 Table). This signature was present despite the lack of functional A3B due to both cell lines being homozygous for the 29.5kb A3B deletion polymorphism [35]. We therefore assessed the mRNA expression level of all seven APOBEC3 family members in SKBR3 and AU565 by qRT-PCR using previously published primers displaying high specificity for individual APOBEC3 family members [36], to identify candidate APOBECs that may be responsible for the observed mutation signature. As previously reported [22, 37] and consistent with both SKBR3 and AU565 being A3B-null, no A3B mRNA was detected in these cell lines. The remaining six APOBEC3 genes had detectable mRNA expression (Fig 1B and S2 Table) including A3A, which is often reported to have undetectable expression in BRCA lines [22, 23]. Remarkably, A3A displayed expression comparable to the remaining APOBEC3 family members, including A3H, which was previously suggested to act as a BRCA mutator in the absence of A3B [23]. The relatively high expression of A3A suggested that it may generate the APOBEC mutation signature in SKBR3 and AU565.

### APOBEC3A is the primary source of cytidine deamination activity in APOBEC3B null AU565 and SKBR3 cell lines

To ascertain if A3A could act as a mutator in cultured BRCA cells, we evaluated cytidine deaminase activity from whole-cell extracts generated from the AU565 and SKBR3 cell lines. We conducted deamination assays with either a 5’ Cy5-labeled linear oligonucleotide substrate (containing a single TCA sequence) or a 5’ Cy5-labeled oligonucleotide that self-anneals into an 11 bp stem and 4 nucleotide loop hairpin structure containing a single TCA trinucleotide in the loop. The sequence of this hairpin substrate was previously identified as a hotspot of APOBEC mutagenesis in breast cancers [16], likely due to A3A’s affinity for hairpin structures [38, 39] because the enzyme bind U-shaped ssDNAs [38, 40]. Cytidine deaminase activity at the TCA sequence in these substrates creates a deoxyuridine base, which can subsequently be converted to a heat liable abasic site by uracil-DNA glycosylase activity (Fig 1C). This enables measurement of cytidine deaminase activity by resolving the substrate from the shorter cleavage product on a denaturing polyacrylamide gel. A3B purified from HEK293T cells displayed comparable cytidine deaminase activity at cytidines in this stem loop compared to those in linear ssDNA (S1 Fig), while purified A3A moderately favored the hairpin forming substrate. Our results indicating that A3A is ~2-fold more active on hairpin structures than ssDNA differ from recently published results [39], which may indicate additional sequence context may modulate the efficiency that APOBECs act on structured DNA. We used the linear and hairpin substrates to initially compare deamination activity in extracts from AU565 or a clonal AU565 cell line containing a compound disruption of three alleles of the *APOBEC3A* gene introduced by CRISPR/Cas9 editing (S2 Fig). Both substrates were deaminated and cleaved in AU565 extracts, whereas no cleavage product was discernable in A3A-deleted AU565 extracts (Fig 1D). To confirm that substrate cleavage was mediated through A3A cytidine deaminase activity, we used lentiviral vectors to generate additional AU565 cell populations expressing an inhibitor of Ung2, Uracil Glycosylase Inhibitor (UGI) [41], or a matched empty-vector control in combination with expression of a scramble-control or A3A transcript-targeting shRNA that specifically reduced A3A mRNA expression as measured by qRT-PCR (Fig 1D). Extracts from cell populations expressing both the scramble shRNA control and the empty-vector control exhibited cleavage of the oligonucleotides that was abolished by UGI expression, which demonstrates cleavage is dependent on cytidine deaminase activity. Moreover, shRNA-mediated reduction of A3A-expression likewise inhibited substrate cleavage, strongly indicating that A3A is the predominant source of cytidine deamination activity in the AU565 BRCA cell line (Fig 1D). Similar requirements of A3A-expression and uninhibited Ung2 activity for both linear and hairpin substrate cleavage were observed in SKBR3 cells combinatorically transduced with empty-vector control, UGI expression vector, scramble shRNA control, and A3A-targeting shRNA (Fig 1E). To determine whether A3A was contributing cytidine deaminase activity solely in the absence of A3B and A3H haplotype I, we genotyped the A3H gene within AU565 and SKBR3. We found that these lines contain at least one A3H haplotype I allele (S3 Table), indicating that A3A is responsible for the majority of cytidine deaminase activity in these A3B-deficient cell lines, even in the presence of A3H haplotype I.

### APOBEC3A is the only APOBEC3 family member with expression that correlates with APOBEC-induced mutation levels in BRCA cell lines

**Fig 2:**
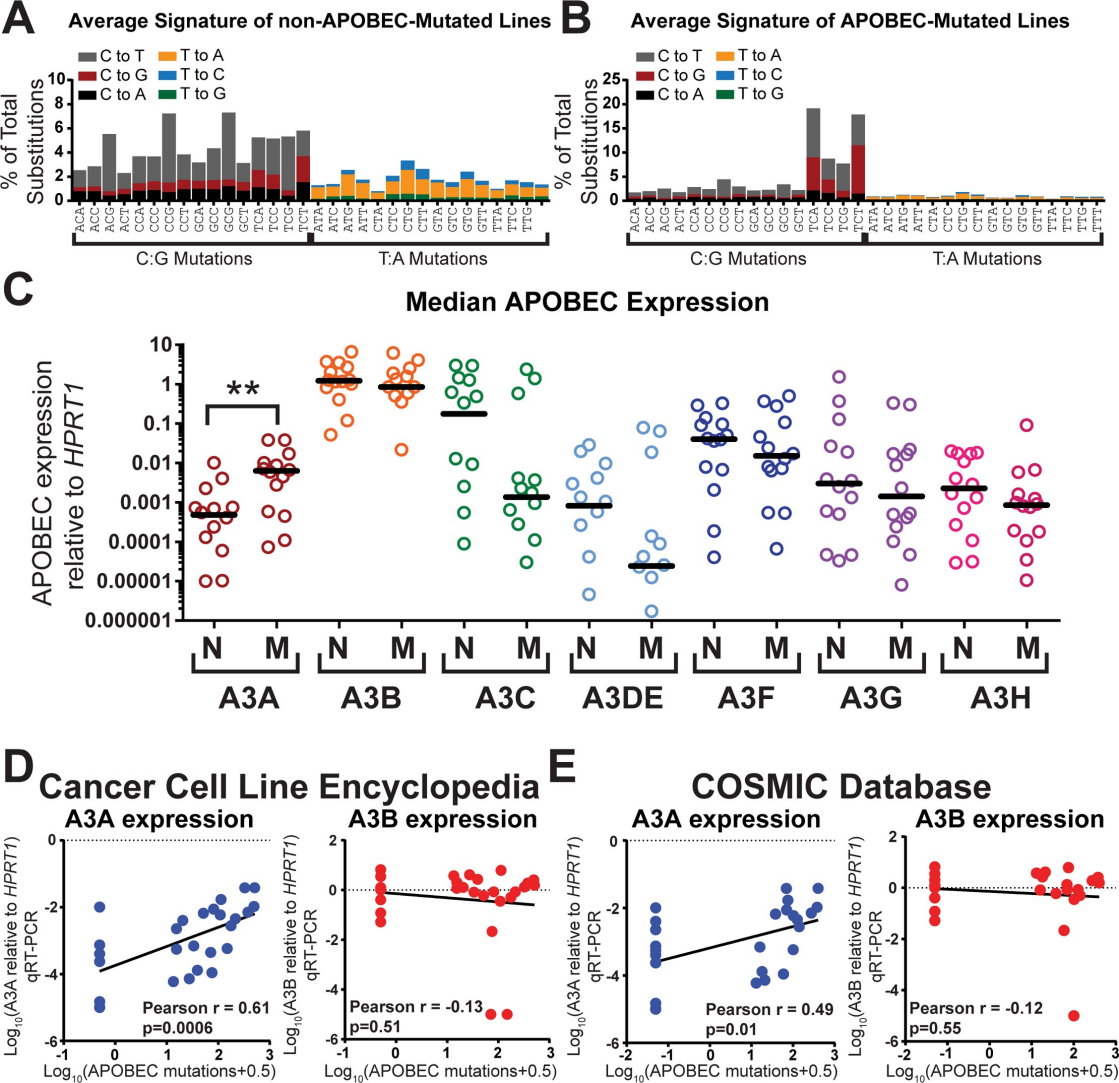
APOBEC3A mRNA transcript levels correlate with the extent of APOBEC-induced mutations in BRCA cell lines. The average mutation profiles of (A) 14 non-APOBEC-mutated and (B) 14 APOBEC-mutated BRCA cell lines. Specific cell lines in each category are defined in S2 Table. (C) mRNA expression level of individual APOBEC3 family members relative to *HPRT1* expression was measured by qRT-PCR in non-APOBEC-mutated (N) and APOBEC-mutated (M) BRCA cell lines. Similar results were obtained comparing APOBEC expression to *TBP* (S2 Table). Each circle represents the mean of 3 replicate measurements for an individual cell line. Horizontal bars indicate the median expression for each APOBEC3 family member among the non-APOBEC-mutated or APOBEC-mutated cell lines. Data points corresponding to cell lines without detectable expression of individual APOBECs are not shown on the graph but are included in the calculation of the median. Statistical significance for differences in the expression of a given APOBEC family member between non-APOBEC-mutated and APOBEC-mutated lines was assessed by Mann-Whitney Summed Rank test. ** indicates p = 0.0067. Correlations between A3A expression (blue dots) or A3B expression (red dots) measured by qRT-PCR and the minimum estimate of APOBEC-induced mutations for each of the 28 BRCA cell lines were determined by a Pearson correlation test using mutation lists obtained from (D) the Cancer Cell Line Encyclopedia and (E) the Catalogue of Somatic Mutations in Cancer (COSMIC).

Given our results that cytidine deaminase activity in APOBEC-mutated cell lines SKBR3 and AU565 was primarily A3A-dependent, we decided to examine the possibility that A3A may play a greater role in APOBEC-catalyzed mutagenesis than previously thought. We began by classifying a panel of 28 BRCA cell lines as either APOBEC-mutagenized or not-APOBEC-mutagenized based on an over-representation of the APOBEC mutation signature as previously described [1]. Non-APOBEC-mutated cell lines displayed an average mutational signature profile consistent with deamination of methylated CpGs [3] (Fig 2A). In contrast, the average signature in those cell lines classified as APOBEC-mutated (Fig 2B), displayed the characteristic APOBEC mutation pattern: a predominance of mutations in the TC dinucleotide, the majority of which are C to G and C to T substitutions at the TCW trinucleotide [3]. We next measured the expression of all the APOBEC3 family members in these cell lines by qRT-PCR (Fig 2C and S2 Table). Consistent with previous reports [1, 2, 22, 37], we found that A3B was expressed at high levels compared to the other APOBEC3s. However, this high expression level existed in both APOBEC-mutagenized and non-APOBEC-mutagenized cell lines, indicating that elevated A3B mRNA levels may not be directly responsible for APOBEC-induced mutagenesis as previously suggested [1, 2, 22]. However, we are unable to exclude that elevated A3B protein level independent of changes in A3B mRNA levels may impact APOBEC mutagenesis. A median 13.1-fold higher A3A mRNA expression level was observed in the APOBEC-mutated BRCA lines compared to non-APOBEC-mutated lines (p = 0.0067 by Mann-Whitney). No other APOBEC3 family member displayed a significant difference in expression level between groups, suggesting A3A may be a major contributor to APOBEC mutagenesis in many BRCA cell lines. Directly plotting A3A expression against either the minimum estimate number of APOBEC-signature mutations or the number of COSMIC Signature 2 and 13 mutations in the 28 BRCA cell lines (mutation lists obtained from the Cancer Cell Line Encyclopedia) produced strong positive correlations (Pearson r = 0.61, p = 0.0006; Fig 2D and Pearson r = 0.70, p<0.0001 S3 Fig, respectively). However, no statistically significant correlation was detected between the abundance of A3B mRNA with the number of APOBEC-signature mutations in these lines. Similar correlations between A3A expression and either APOBEC-signature mutations or Signature 2 and 13 mutations was observed for mutation data from 27 BRCA cell lines obtained from the COSMIC database (Pearson r = 0.49, p = 0.01; Fig 2E and Pearson r = 0.60, p = 0.0009 S3 Fig, respectively). While these results are correlative and we are therefore unable to exclude the possibility of co-correlates that may underlie the association of A3A expression with the number of APOBEC-induced mutations, the simplest interpretation of these results is that A3A may be a predominant source of APOBEC-signature mutations in BRCA cell lines, even those expressing high levels of A3B.

### APOBEC3A contributes to APOBEC-induced deamination activity in BRCA cell lines with high levels of APOBEC3B mRNA

**Fig 3:**
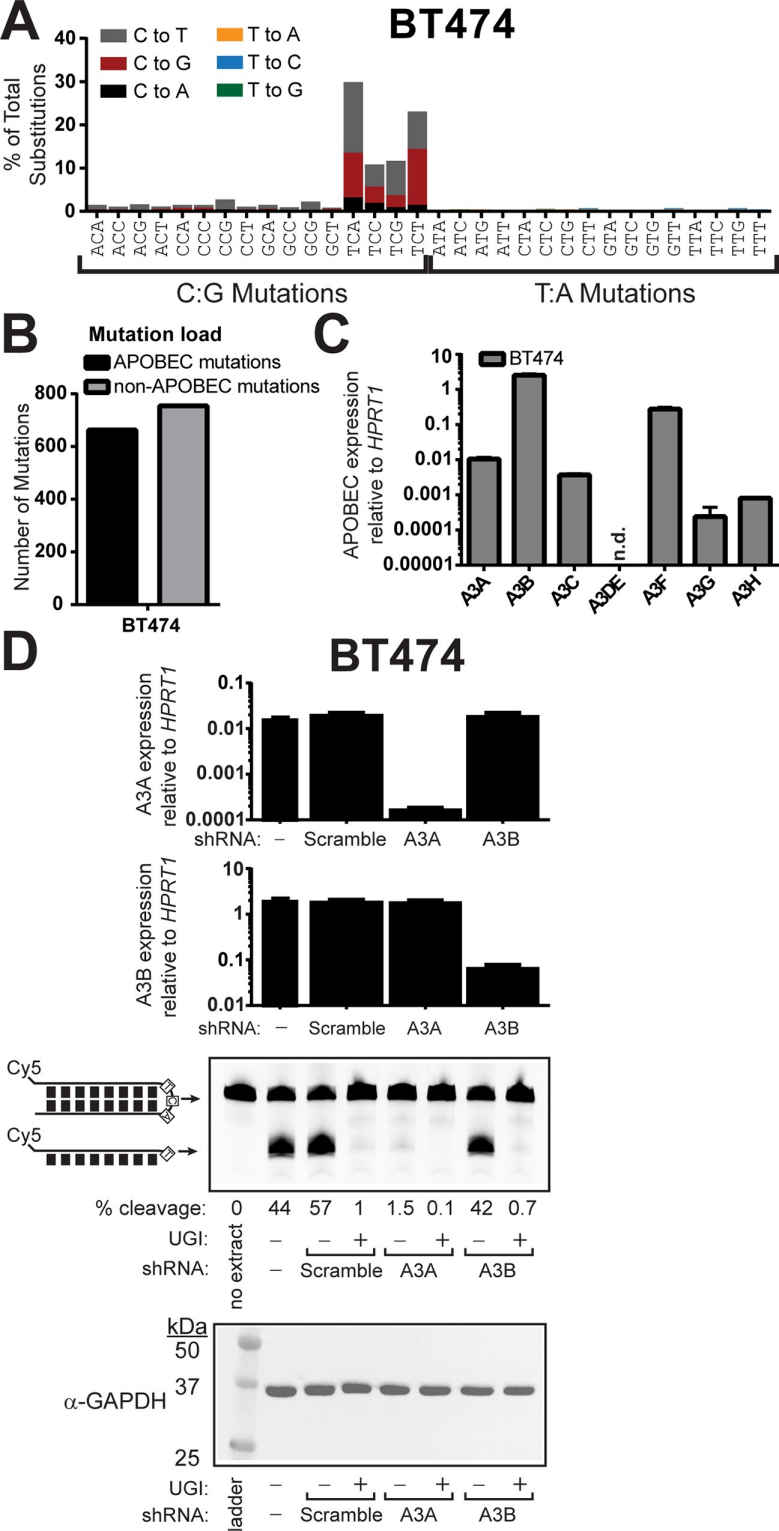
APOBEC3A is the predominant cytidine deaminase in BT474 cells expressing APOBEC3B. (A) The mutation profile of BT474 cells. (B) The number of APOBEC-induced (black bars) and non-APOBEC-induced (grey bars) mutations in BT474 cells. (C) mRNA expression levels of individual APOBEC3 family members relative to *HPRT1* expression in BT474 as measured by qRT-PCR. Bars indicate the mean values of 3 replicate measurements. Error bars indicate the standard error of the mean of these measurements. n.d. indicates “not detected.” (D) *In vitro* cytidine deaminase assay (conducted similarly to Fig 1D and 1E) of whole-cell extracts generated BT474 cells or BT474 cells transduced with lentiviral vectors to express scramble control, A3A-targeting, or A3B targeting shRNAs. Deaminase reactions were supplemented with either 2 units UGI (NEB #M0281S) or an equal volume of 50% glycerol. Specificity of each shRNA was confirmed by qRT-PCR, and equal protein amounts used in deaminase assays were verified by α-GAPDH western.

We quantified the contribution of A3A and A3B to cytidine deaminase activity in the APOBEC-mutated cell lines BT474, CAMA-1, and MDA-MB-453, which respectively express 243-, 182-, and 269-fold higher A3B than A3A by qRT-PCR (S2 Table) and contain at least one A3H haplotype I allele each (S3 Table). Among the cell lines we analyzed, BT474 has the highest total number and largest proportion of APOBEC mutations (Fig 3A and 3B and S1 Table) and has detectable mRNA expression of all APOBEC3 enzymes, except APOBEC3DE (Fig 3C and S2 Table). We generated stable BT474 cell populations expressing a scramble-control shRNA, A3A-targeting shRNA, or A3B-targeting shRNA. Initial attempts with multiple A3B-targeting shRNAs, including shRNA TRCN0000140546 (A3B-1 in S4 Fig), used in [22], revealed non-specific 4- to 13.8-fold knockdown of A3A (depending on cell line assessed) in addition to decreasing A3B expression, as intended. We therefore engineered additional shRNAs that respectively decreased A3A and A3B 58- and 28-fold, without significant impact on other APOBEC3 family members (Fig 3D, S4 Fig, and S2 Table). As observed in AU565 and SKBR3 extracts, whole-cell extracts from parental BT474 cells and BT474 cells expressing the scrambled shRNA efficiently cleaved the hairpin DNA substrate. The addition of UGI to the reaction inhibited substrate cleavage, confirming that it was mediated through cytidine deaminase activity (Fig 3D). Despite A3B mRNA transcript levels being ~243-fold higher than A3A, knockdown of A3A expression surprisingly eliminated cytidine deaminase activity, while knockdown of A3B expression had no effect, which indicates that A3A is the dominant cytidine deaminase in BT474 cells. To ensure the lack of detectable A3B activity was not due to our hairpin substrate containing the A3A-favored YTCA tetranucleotide target sequence, we repeated this experiment with a substrate containing the A3B-preferred RTCA motif. Even with this optimal substrate for A3B activity, cytidine deaminase activity was nearly eliminated with decreased A3A expression, while 28-fold down-regulation of A3B failed to significantly impact activity (S5 Fig). Similar results were obtained with extracts from CAMA-1 and MDA-MB-453 cells expressing either scrambled shRNA, A3A-targeting shRNA, or A3B-targeting shRNA in which cytidine deaminase activity was almost entirely dependent on A3A expression (S6 Fig). Thus, A3A is a major source of cytidine deaminase activity in all five APOBEC-mutated cell lines we examined, which is a strong indicator that A3A may be largely responsible for APOBEC activity in most BRCA tumors.

### Cellular RNA severely inhibits APOBEC3B, but not APOBEC3A

**Fig 4:**
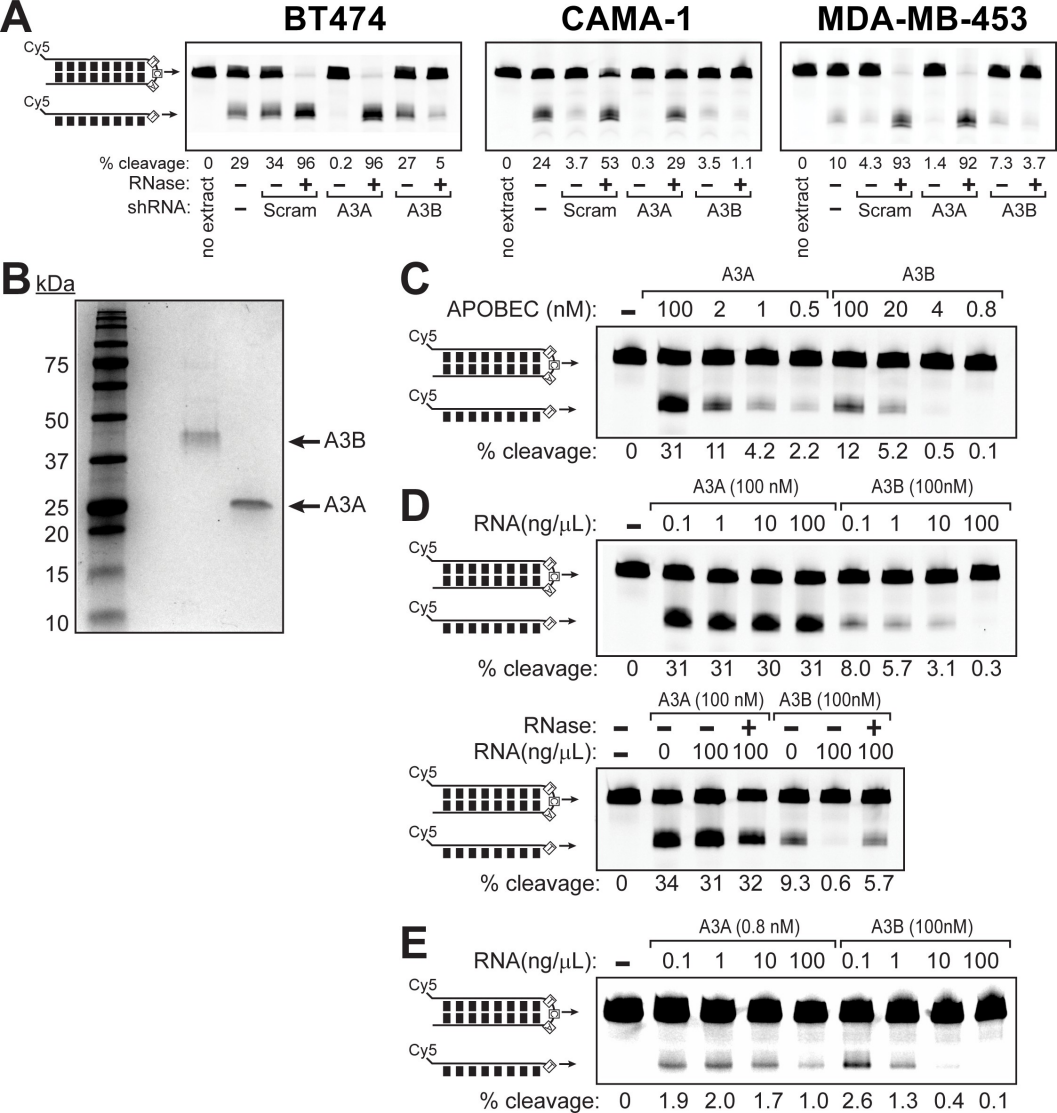
APOBEC3A and APOBEC3B are differentially inhibited by RNA. (A) *In vitro* cytidine deaminase assays conducted similarly to those in Fig 1D and 1E, but using extracts for BT474, CAMA-1 and MDA-MB-453 cells transduced to express scramble (Scram) shRNA controls or A3A- and A3B-targeted shRNAs. Prior to incubating the extracts with the hairpin DNA substrate, extracts where either mock-treated or treated with 2.5 μM RNaseA at 37°C for 30 min. (B) Coomassie stained SDS-PAGE showing the purity of A3A (23 kDa) and A3B (42 kDa) over-expressed in and purified from HEK293T cells. (C) The activity of purified A3A and A3B was compared *in vitro* by incubating 0.25 μM hairpin DNA substrate with decreasing concentrations of A3A or A3B for 30 min at 37°C in the presence of uracil DNA glycosylase. Reactions were heated at 95°C for 10 min in formamide loading buffer prior to resolving on a denaturing polyacrylamide gel. (D) Increasing amounts of total RNA isolated from BT474 cells were added to *in vitro* cytidine deaminase assays containing 100 nM of either A3A or A3B and 0.25 μM of hairpin DNA substrate to assess the relative impact of RNA on A3A and A3B activity. Additionally, reactions containing 100 ng/μL of RNA were either mock treated or treated with RNaseA to ensure inhibition of cytidine deaminase activity was due to interactions with RNA. (E) To assess RNA inhibition of A3A and A3B at similar enzymatic activities, 0.8 nM A3A and 100 nM A3B where incubated with 0.25 μM of hairpin DNA substrate and increasing RNA concentrations. Samples in (D) and (E) were processed as in (C).

The dominance of A3A in providing cytidine deaminase activity in APOBEC-mutated BRCA cell lines was surprising given the protein’s relatively low expression and prior results indicating A3B played this role [22, 42]. For the majority of cell lines tested, the previous cytidine deaminase assays that support a greater role for A3B in BRCA were conducted in the presence of 156 μg of RNaseA [42], while our experiments were performed without the removal of RNA from the extracts. Hence, we hypothesized that the presence of RNA in our cell extracts could account for the difference in results. Many APOBEC cytidine deaminases, including A3B, bind RNA [43–48], which frequently results in inhibition of cytidine deaminase activity. We therefore repeated our *in vitro* cytidine deaminase assay using BT474, CAMA-1, and MDA-MB-453 extracts that were treated with RNaseA to remove residual RNA. As expected, addition of RNaseA greatly increased overall cytidine deaminase activity in extracts from all three cell lines. Moreover, this increase was completely abolished by shRNA mediated knockdown of A3B (Fig 4A), indicating that the presence of RNA strongly inhibits A3B activity.

Taken together, our cytidine deaminase activity assays in breast cancer cell extracts, suggests that A3A is the dominant cytidine deaminase active on hairpin substrates in the presence of RNA, while in the absence of RNA, A3B dominates. We verified this association by measuring the deamination of our hairpin substrate in cell extracts from 10 breast cancer cell lines that were either untreated or treated with RNAseA and comparing the activity to either A3A or A3B mRNA expression level (S7 Fig). This comparison revealed a strong correlation (Pearson r = 0.72 and p = 0.018) between A3A mRNA expression and cytidine deaminase activity in extracts that were not treated with RNAseA. In contrast, A3B mRNA expression strongly correlated with cytidine deaminase activity in extracts that were RNAseA treated (Pearson r = 0.89 and p = 0.0007). These results support that in BRCA cell lines A3A and A3B mRNA levels are strong surrogate measure of the protein level and cellular activities of the respective enzymes.

Previous work postulated that RNA binding to the N-terminal cytidine deaminase domain (CDD) of A3B may function as a negative regulator of A3B activity in human cells [48]. While A3A and the C-terminal CDD of A3B are ~90% identical, A3A lacks the N-terminal domain of A3B [4]. Hence, RNA-binding may differentially regulate the activity of A3A and A3B. To test this hypothesis, we purified full-length, strep-tagged A3A and A3B proteins from human HEK293T cells (Fig 4B) and assessed each protein’s sensitivity to RNA inhibition *in vitro*. Measurement of cytidine deaminase activity toward our hairpin DNA substrate indicated that A3A was 3-fold more active than A3B when incubated at near stoichiometric concentrations compared to substrate. However, A3A retained most of its activity even at a 1000:1 substrate to enzyme ratio, while A3B activity quickly decreased once diluted to sub-stoichiometric concentrations. This result indicates that, as when purified from *E*. *coli* [49], A3A from human cells is significantly more active (Fig 4C) than full-length A3B. Additionally, incubation of 500 nM A3A with up to 2000 ng/μL total RNA isolated from BT474 cells failed to significantly inhibit the enzyme, while as little as 1 ng/μL of total RNA began inhibiting A3B activity (Fig 4D and S8 Fig). A3B’s greater inhibition by RNA than A3A was apparent even when the amounts of these enzymes were reduced and scaled to similar activity levels (Fig 4E). A3B, but not A3A, was inhibited regardless of whether RNA was isolated from total cells, nuclei, or the cytoplasm (S9 Fig). In the presence of 100 ng/μl RNA, A3A is >100 fold more active than A3B. Given the typical human cell contains ~10 pg of RNA [50], the diameter of BRCA cells fall in the range of 12.07 to 15.08 μm [51], and the average concentration of RNA is ~3.5 fold higher in the cytoplasm than nucleus of mammalian cells [52], a minimum estimate of the RNA concentration within the nucleus of BRCA cells is ~1616 ng/μL. Considering our results that A3B cytidine deaminase activity is nearly undetectable in the presence of 100 ng/μL RNA, it is likely that A3B activity is severely inhibited by RNA in cells, enabling A3A to be the primary cytidine deaminase despite its lower expression level. A recent study determined that A3H, which has also been suggested to be a BRCA tumor mutator [23], is also inhibited by RNA binding due to a patch of five basic amino acids within the A3H CCD [45]. The homologous region of A3A only contains two basic amino acids in this region (S10 Fig) providing further structural insights into A3A’s unique resistance to RNA. Hence, negative regulation of both A3B and A3H deamination activity by RNA likely reduces their ability to damage genomic DNA as compared to A3A.

### APOBEC3A expression correlates with APOBEC-induced mutagenesis in primary BRCA tumors

**Fig 5:**
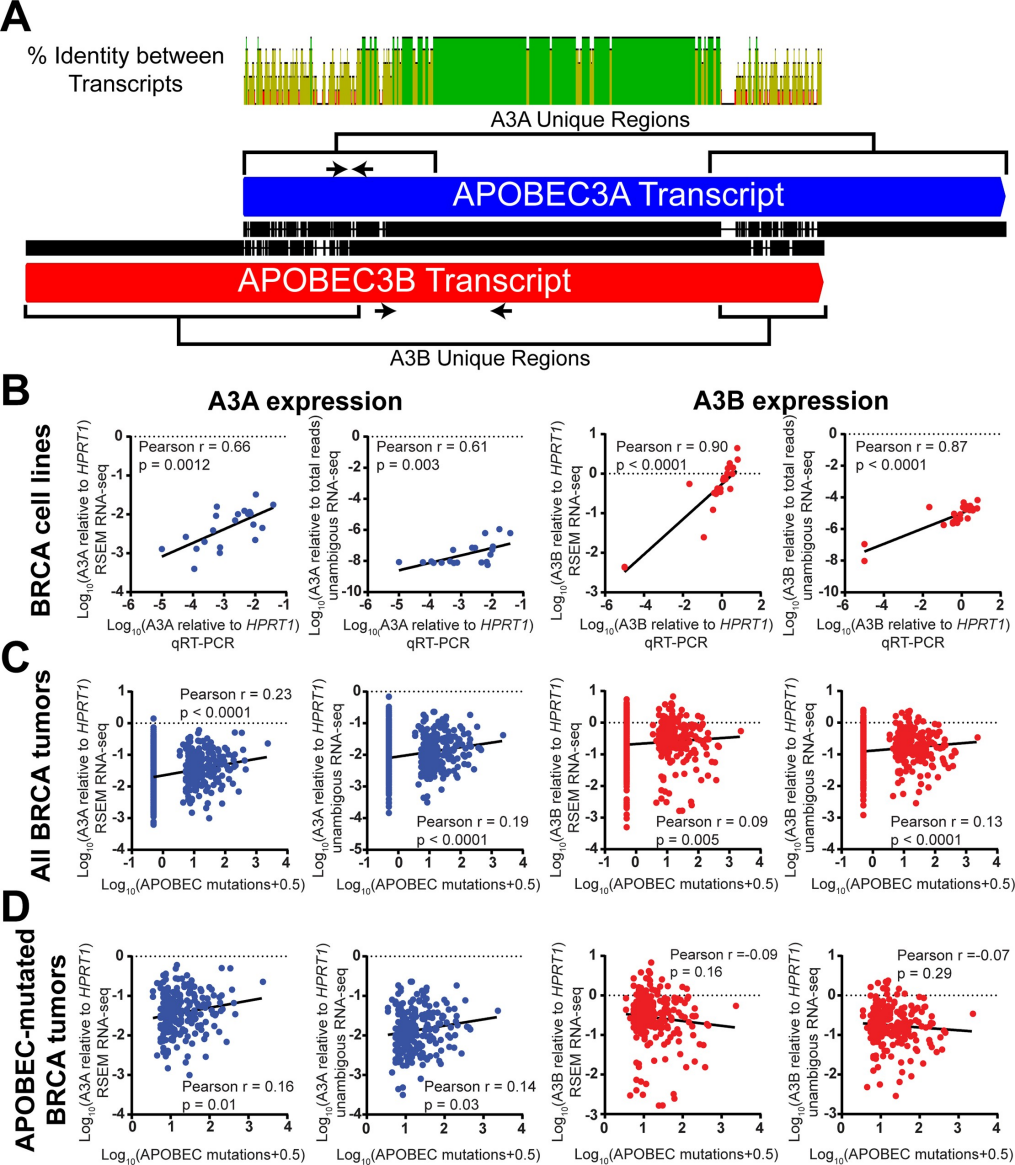
APOBEC3A expression correlates with the abundance of APOBEC-induced mutations in primary BRCA tumors, despite the similarity between APOBEC3A and APOBEC3B transcripts. (A) The pairwise alignment of the A3A (blue) and A3B (red) transcripts shows a central region of high sequence identity with two unique regions in each transcript. Green, yellow, and red indicate 100%, ≥30%, and <30% identity respectively. Arrows indicate the position of qRT-PCR primers. (B) Correlations between qRT-PCR measurements of A3A or A3B mRNA abundance and corresponding RSEM normalized RNA-seq measurements or RNA-seq measurements limited to reads mapping within the defined unique regions of A3A and A3B transcripts (unambiguous RNA-seq) for BRCA cell lines were assessed by Pearson correlation test. A3A and A3B expression measured by RSEM normalized RNA-seq or unambiguous RNA-seq was compared to the minimum estimate of APOBEC-induced mutations in (C) 973 TCGA sequenced primary BRCA tumors or (D) a subset of 229 APOBEC-mutated TCGA sequenced primary BRCA tumors by Pearson correlation test.

Because our data implicates A3A as a major source of cytidine deaminase activity and mutagenesis in many BRCA cell lines, we sought to determine whether A3A also contributed to the mutagenesis of primary BRCA tumors. Prior analyses had observed a positive correlation for both A3A and A3B mRNA expression levels with the number of APOBEC-induced mutations in 483 primary breast cancers characterized by TCGA [1]. However, it was postulated that the observed correlation between A3A expression and APOBEC-induced mutation load could stem from mis-mapping of RNA-seq reads from A3B’s C-terminal cytidine deaminase domain (CDD) to the highly homologous CDD of the A3A [23]. We therefore assessed the accuracy of RNA-seq in determining the expression levels of A3A and A3B by comparing transcript measurements from BRCA cell lines using qRT-PCR, RSEM normalized RNA-seq, and RNA-seq only utilizing reads that mapped to the unique regions of A3A and A3B transcripts (Fig 5A). Comparison of qRT-PCR measurements to RSEM normalized RNA-seq (obtained from the Cancer Cell Encyclopedia) for 21 BRCA cell lines revealed a strong positive correlation between the methods for both A3A transcript and A3B transcripts (Fig 5B, S1 Table, and S2 Table), indicating that RNA-seq based analyses are likely accurate in their discrimination between these genes. Further supporting this, re-analysis of A3A and A3B transcript levels only utilizing RNA-seq reads mapping to unique regions of A3A and A3B (referred to as unambiguous RNA-seq) produced similarly strong positive correlations with both qRT-PCR based measurements (Fig 5B) and RSEM normalized RNA-seq values (S11 Fig). Comparison of the A3A and A3B expression levels for 1207 TCGA-characterized BRCA tumors measured by unambiguous RNA-seq and RSEM-normalized RNA-seq, likewise showed a very strong positive correlation (S11 Fig; Pearson r = 0.91 and 0.96, for A3A and A3B respectively), again indicating that RSEM-normalized RNA-seq accurately depicts both A3A and A3B expression. Comparison of A3A and A3B expression to APOBEC-induced mutation load (by either the minimal estimate of APOBEC-signature mutations or the number of COSMIC Signature 2 and 13 mutations) for 973 of these tumors recapitulated previously published results depicting a modest but highly significant positive correlation for both genes. Assuming that the correlations of A3A and A3B mRNA levels with mutagenesis are independent of other unknown correlates and indicative of elevated protein level of these enzymes, these results suggest that both A3A and A3B likely participate in the mutagenesis of some specific samples (Fig 5C and S12 Fig). However, the positive correlation was stronger for A3A expression compared to A3B expression, suggesting that A3A may play a larger role in primary BRCA tumor mutagenesis. Moreover, when restricting the correlation analysis to the 229 APOBEC mutagenized BRCA tumors, we still observed a significant correlation between A3A expression and APOBEC mutation load (Pearson rho = 0.16, p = 0.01) while no correlation was evident for A3B expression (Fig 5D). This result indicates that A3A expression may directly influence the extent of mutagenesis in BRCA, while additional factors in combination with higher A3B expression are needed for A3B-mediated mutagenesis to occur. Similar results were observed with correlation analyses comparing A3A and A3B unambiguous RNA-seq to APOBEC-induced mutation loads (Pearson rho = 0.14, p = 0.03 for A3A expression). A3A and A3B expression have been previously shown to correlate with each other [23] (S13 Fig) and therefore the relationship between elevated A3A expression on the abundance of APOBEC-induced mutation could in theory result from higher A3B expression in the APOBEC-mutated tumors with high A3A expression. We therefore, repeated this analysis with a subset of BRCA tumors where in the lowest quartile for A3B expression. As with the entire BRCA dataset, A3A expression still positively correlated with APOBEC-induced mutation load despite limited A3B expression (S13 Fig; Pearson rho = 0.23, p = 0.0003 and Pearson rho = 0.33, p = 0.03 for all low-A3B BRCA tumors and APOBEC-mutated low-A3B BRCA tumors, respectively). Conversely, no correlation was detected between A3B expression and APOBEC-signature mutations in tumors with low A3A expression. Thus, this comparative analysis of APOBEC expression data supports a major role for A3A in breast cancer mutagenesis independent of A3B.

We note that the strength of the correlation between A3A and APOBEC mutagenesis in primary BRCA tumors is significantly less than observed among BRCA cell lines. Multiple differences in the sample sets may explain this discrepancy, including the existence of modulators of APOBEC-driven mutagenesis and episodic mutation events that are specific to the tumor environment. Furthermore, tumors samples contain subpopulations of tumors cells and contamination with non-tumor cells that increases cellular heterogeneity, which adversely affects correlation analyses for RNA-seq expression data. Alternatively, failure of APOBEC mRNA expression levels to accurately predict the protein level of these enzymes or the presence of additional factors beyond A3A expression level that influence the amount of APOBEC-induce mutation could also weaken the correlation of A3A mRNA level with mutation in primary tumors. Our initial analysis of A3A and A3B activity in BRCA cell lines (S7 Fig) supports that A3A and A3B mRNA levels are strong indicators of their respective enzyme’s activity, however, whether this correlation extends to primary tumors is unknown. We cannot exclude however the possibility of other unknown tumor specific characteristics, such as differences in the level of Ung2 activity, that may also influence the correlation between A3A expression and APOBEC mutation load.

### APOBEC3A expression correlates with APOBEC-induced mutagenesis in tumors from multiple cancer types

**Fig 6:**
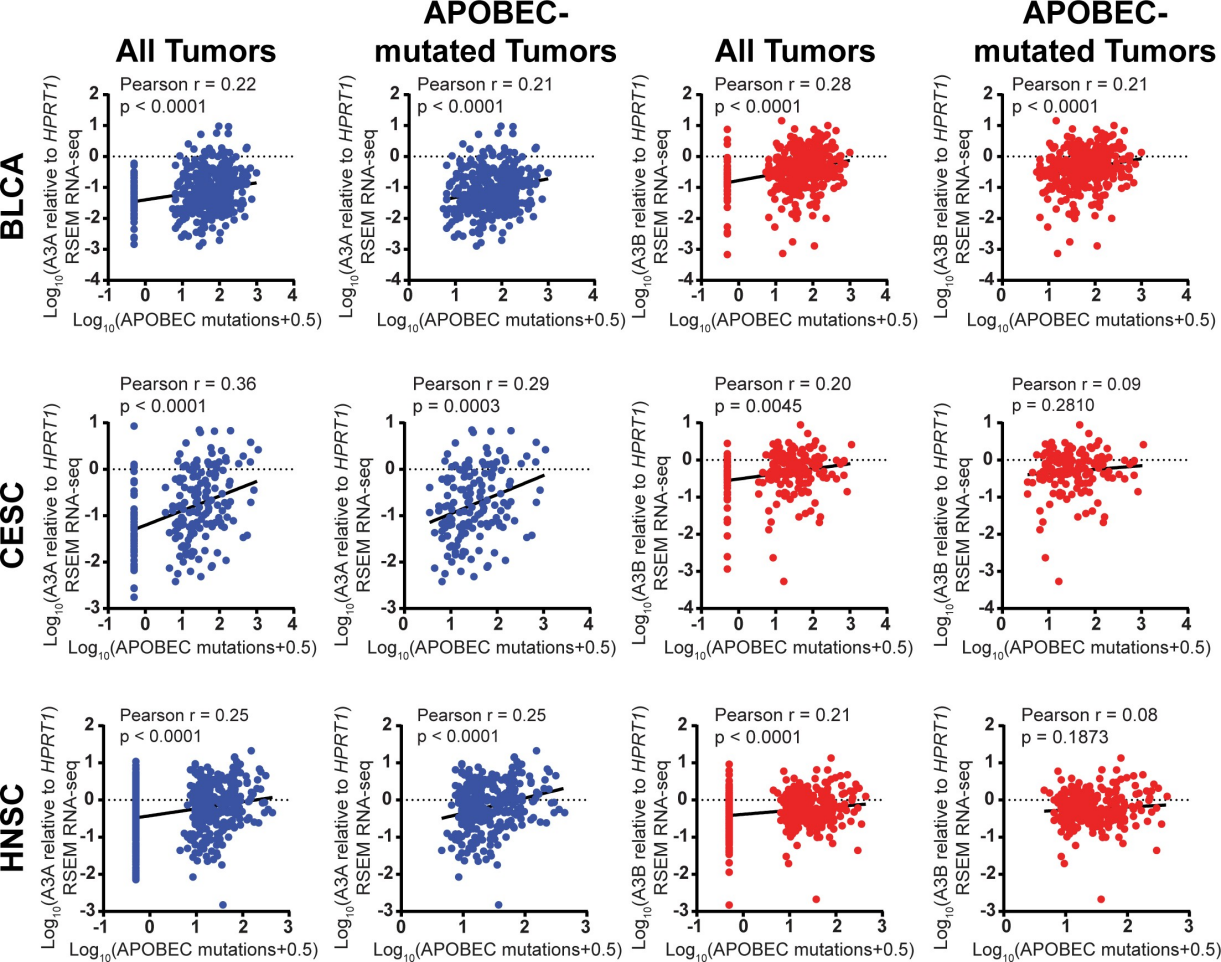
Correlation of A3A expression with APOBEC-induced mutations in multiple cancer types. RSEM normalized RNA-seq values for A3A (blue) and A3B (red) were obtained for 410 bladder (BLCA), 197 cervical (CESC), and 549 head and neck (HNSC) cancers assessed by the Cancer Genome Atlas. Expression of each APOBEC was normalized to *HPRT1* and compared to the minimum estimate of APOBEC-induced mutations in the corresponding samples. A3A expression strongly correlates with mutagenesis in each tumor type by Pearson correlation analysis even when only evaluating APOBEC mutated tumors.

We next decided to evaluate whether A3A expression also predicts the amount of APOBEC-induced mutation in other cancer types to assess whether A3A may significantly contribute to mutagenesis beyond BRCA. We therefore downloaded RSEM-normalized RNA-seq values for A3A, A3B, and *HPRT1* from 410 bladder cancers (BLCA), 197 cervical cancers (CESC), and 549 head and neck cancers (HNSC) from the TCGA. The comparison of A3A and A3B expression to the abundance of APOBEC-induced mutations showed significant correlation of both APOBECs with mutagenesis among all assessed tumors (Fig 6, S1 Table, and S2 Table). The extent of correlation was also similar in BLCA and HNSC (Pearson rho = 0.22 for A3A and 0.28 for A3B in BLCA and 0.25 for A3A and 0.21 for A3B in HNSC), while A3A expression correlated more strongly than A3B in CESC (Pearson rho = 0.36 for A3A and 0.20 for A3B). These correlations support A3A as a significant mutator in cancers beyond BRCA. As with BRCA, however, only A3A expression continued to correlate with the amount of APOBEC-induced mutation among APOBEC-mutagenized tumors in all three cancer types, which indicates that A3A expression itself likely determines the extent of mutagenesis in these tumors. In contrast, A3B expression only correlated with APOBEC-induced mutation in BLCA APOBEC-mutated tumors. Therefore, A3B expression appears to be higher in APOBEC-mutagenized CESC and HNSC tumors, but additional factors are likely required to enable A3B-mediated mutation in these cancers.

## Discussion

Previous studies have implicated multiple APOBEC cytidine deaminases in establishing the APOBEC mutation signature within breast cancers [22, 23, 33]. Prior to our study, seminal work by Harris and colleagues had provided the only experimental evidence of an endogenously expressed APOBEC3 protein contributing to cellular cytidine deamination activity and mutagenesis [22]. They utilized shRNA-mediated knockdown of A3B in specific BRCA cell lines and observed lower amounts of cytidine deaminase activity in whole cell extracts, lower levels of genomic dU, and decreased mutation of an HSV-TK mutation reporter. Two additional reports utilized bioinformatics analyses to suggest A3H haplotype I [23] and A3A [33] as plausible APOBECs additionally contributing to cancer mutagenesis. Harris and colleagues found that some A3B deleted BRCA tumors still maintained APOBEC signature mutations. Careful examination of the A3H haplotypes of the assessed tumors revealed that all the APOBEC mutated, A3B-deleted tumors had at least one A3H haplotype I allele, suggesting that A3H haplotype I may be contributing to mutagenesis [23]. However, no experimental evidence of endogenously expressed A3H haplotype I activity was provided. In addition, Gordenin and colleagues determined that A3A may contribute significantly to cancer mutagenesis through the assessment of extended mutation signatures specific to A3A and A3B [33]. By expressing each enzyme in yeast and analyzing the resulting mutation spectra of the *CAN1* forward mutation reporter, they determined that A3A and A3B have differential preference at the -2 nucleotide of the target motif, where A3A prefers YTCA while A3B prefers RTCA. Analysis of thousands of whole genome sequenced tumors determined that the majority of APOBEC-mutagenized tumors contained mutations over-represented in YTCA sequences compared to RTCA, suggesting that A3A may be a major cancer mutator, though similar to the Harris study on A3H haplotype I, experimental evidence supporting detectable cytidine deaminase activity of endogenously expressed A3A was lacking. In fact, several groups recently argued that A3A has little to no role in cancer mutagenesis [23] because; (1) A3B and A3A can deaminate each other’s preferred motifs and these extended mutation signatures were generated from a yeast system expressing the human APOBEC proteins, which may not fully recapitulate the substrate preferences of individual APOBECs in human cells, (2) A3A is often lowly expressed and (3) cell imaging experiments have indicated that endogenous A3A is primarily cytosolic in localization [53].Thus, A3A seemed ill-positioned to induce mutations in chromosomal DNA, especially considering the lack of prior evidence of detectable endogenous A3A cytidine deaminase activity. Taken together, previous studies paint an unclear picture as to the importance of individual APOBECs to BRCA mutagenesis and there are several strongly held, prevalent, opposing opinions as to the relative contribution of A3A, A3B, and A3H haplotype I.

Our data is consistent with A3A being responsible for APOBEC deaminase-derived mutations in most BRCA tumors. Here, we show that APOBEC-mutagenized BRCA cell lines have higher A3A expression levels compared to non-APOBEC mutagenized lines and that A3A expression alone correlates with the number of APOBEC-signature mutations. Moreover, we demonstrate in whole-cell extracts from five APOBEC-mutagenized cell lines that A3A provides the majority of cytidine deaminase activity, which indicates A3A is ubiquitously present and active in APOBEC-mutated BRCA cells. In the presence of cellular RNA, A3A contributes significantly to cytidine deaminase activity, even from extracts from BRCA cell lines with elevated A3B expression and containing A3H haplotype I, indicating that in some cellular contexts, A3A is the dominant APOBEC present in the cell. The prevalent contribution of A3A to cellular cytidine deaminase activity, despite A3B mRNA transcripts being ~2-orders of magnitude higher than A3A, is likely due in part to the higher intrinsic activity of A3A compared to A3B, as well as strong inhibition of both purified and cellular A3B by RNA. A3A expression likely contributes to mutagenesis within primary breast cancers as A3A mRNA transcript levels positively correlate with APOBEC-induced mutation load, when expression is measured by RNA-seq or our novel unambiguous RNA-seq. In contrast to A3B expression, A3A transcript levels also correlated with APOBEC-induced mutation load when limiting the analysis to APOBEC-mutated tumors. These results provide experimental and computational evidence supporting our conclusion that elevated A3A expression is likely responsible most APOBEC-induced mutations in breast cancer.

We also found that levels of A3A mRNA transcripts have a stronger linear correlation with APOBEC-induced mutagenesis than does A3B transcript levels among BRCA cell lines and for primary BRCA, CESC, and HNSC tumors indicating that A3A likely contributes significantly to APOBEC-induced mutation across multiple cancer types. While one previous report using immunohistochemistry suggested that endogenous A3A was localized primarily within the cytoplasm (which would make A3A-induced mutagenesis difficult) [53], multiple reports indicate a pan-cellular localization of A3A [25, 53, 54] due to passive diffusion of this small 22 kDa protein through the nuclear membrane [53]. This existence of some A3A within the nucleus is supported by other data showing that exogenous expression of A3A, even at levels less than seen in interferon activated CD14+ cells, causes cytotoxicity and DNA damage [53–55]. This genomic DNA damage has since been determined to be replication-dependent in human cells, consistent with the pattern of APOBEC-induced mutation in tumors, [20, 55]. Upregulation of endogenous A3A also can cause DNA damage in some cell lines [31], indicating that A3A activity can occur in the nucleus. A3A expression has been reported to be elevated in response to the A3B germline deletion polymorphism both in cell line systems [35] and in oral cancers carrying the polymorphism [56], suggesting that elevated endogenous A3A expression likely also contributes to genetic instability in cancer cells.

Modest correlation of A3B expression with the amount of APOBEC-induced mutations in BRCA, BLCA, CESC, and HSNC cancers indicates that along with A3A, A3B likely contributes to the mutagenesis of some human tumors. This is supported by the analysis of the -2 nucleotide preference in whole genome sequenced tumors, which identified a subset of BRCA tumors that were enriched for mutations within the A3B preferred RTCA motif [33]. However, these tumors were fewer in number and contained fewer mutations, which is consistent with our results showing A3B has much lower activity compared to A3A at near cellular RNA levels. Germline polymorphisms in the A3B gene have been linked to higher levels of the protein and elevated amounts of mutagenesis in bladder cancers [57]. Correspondingly, other common polymorphisms in the *APOBEC3* locus associate with reduced A3B expression and RTCA mutagenesis in pan-cancer analyses [58]. These specific associations between germline sequence in the *APOBEC3B* region and modulation of the APOBEC mutation signature in cancers provide evidence for its potential as a cancer mutator and are validated by experimental evidence indicating that reduction of A3B can be anti-mutagenic [22] and recent determination that endogenous A3B is responsible for induced kataegis events in RPE-1 cells [59]. The discovery that several APOBEC-targeting shRNAs (including one used in the seminal Burns *et al*. manuscript [22] to decrease A3B expression) also significantly decrease A3A expression makes it difficult to ascertain from some reports whether the observed phenotypes are the result of A3A or A3B down-regulation. Moreover, A3A expression correlates more strongly with APOBEC-induced mutagenesis in each of these cancer types and, unlike A3B expression, continues to correlate when solely assessing APOBEC-mutagenized tumors, indicating that the level of A3A expression is one of the primary factors dictating the extent of mutation in these cells.

Our work suggests that A3A may be a better candidate than A3H haplotype I in causing the APOBEC mutation signature in A3B deleted breast cancers. In contrast to A3A, both A3B and A3H haplotype I [44, 45] are strongly inhibited by RNA indicating that both enzymes likely require activation for activity in cells, similar to what has been reported for the related APOBECs, AID [60, 61] and APOBEC3G [62]. However, any mechanism for relieving RNA inhibition of A3B or A3H is currently unknown. While inhibition of A3B by RNA is likely relieved by some mechanism, we were unable to detect any A3H haplotype I activity in AU565 or SKBR3 cells (that have at least one A3H haplotype I allele) even when assessing deaminase activity a linear oligonucleotide substrate, which A3H prefers [39]. This observation suggests that A3H haplotype I may maintain insufficient activity in cells to act as a tumor mutator. A3H haplotype I is the most common haplotype in humans, occurring at an allele frequency of ~50% based on prior estimates [30, 63, 64]. Therefore, >98% of tumors are likely to have at least one A3B or A3H haplotype I allele. Consistent with this, the initial implication of A3H haplotype I in cancer mutagenesis is based on the absence of APOBEC signature mutations in three A3B deleted non-A3H haplotype I breast cancers [23]. Since only ~25% of BRCA tumors are APOBEC mutagenized [1], approximately 42% of the time randomly sampling would produce three non-APOBEC-mutated BRCA tumors, making it impossible to conclude lack of A3H-haplotype I is responsible for the absence of the APOBEC mutation signature.

A3A expression significantly correlates with APOBEC-signature mutations in BRCA cell lines and primary tumors, but tumors often significantly deviate from this trend. One possible explanation for variation in the observed frequency of APOBEC signature mutations is the existence of modifiers of APOBEC activity that differ among tumor samples. Identification and characterization of the mechanisms that control APOBEC-activity in tumors and determining the relative contributions of A3A, A3B, and A3H in tumors of different cancer types will be key to facilitating researchers’ and clinicians’ efforts to develop treatments for APOBEC-mutagenized tumors. Our results that RNA binding dramatically inhibits A3B, but not A3A, activity highlights a potential role for RNA binding as key APOBEC regulator. Future studies are needed to determine if A3B is similarly inhibited by RNA in all cellular contexts, or if cellular factors like post-translational modification, alternative splicing, or cell type-specific protein interactions allow A3B to be more active against genomic DNA in the presence of RNA. Other mechanisms that regulate the mutagenic capacity of APOBECs and protect the genome of human cells also likely exist. In addition to its inhibition by RNA, PKA phosphorylation of A3B at residue T214 limits A3B’s ability to bind ssDNA and deaminate the substrate [65], likely providing and additional negative regulator of A3B-induced mutagenesis. Similarly, Tribbles3 has been reported to inhibit A3A in human cells through its mediation of proteasome independent A3A degradation [66], providing one plausible control on aberrant A3A activity. Beyond direct modification of APOBEC proteins, repair of APOBEC-induced lesions or control of ssDNA substrate availability likely also influence the extent of APOBEC-induced mutation. We previously found in yeast that recombination-mediated error-free bypass reduces APOBEC-induced mutagenesis [67] and speculate a similar repair mechanism may exist in human cells and that specific repair capacities in human cells might influence the accumulation of APOBEC-signature mutations in cancer. Furthermore, APOBECs’ ability to access genomic ssDNA may be modulated by the amount of replication stress tumor cells experience due to specific combinations of oncogenes and tumor-suppressor mutations. Variation in the activity of these control mechanisms may affect the accumulation of APOBEC-signature mutations in tumors. Some of these regulatory mechanisms may function transiently within cells and thereby contribute to the episodic occurrence of the APOBEC mutation signature reported in cultured BRCA cells [34]. Currently, the cellular contexts in which these regulatory mechanisms operate as well as how they are modulated during cancer development remain unclear. Characterizing these mechanisms that regulate APOBEC-induced mutagenesis during carcinogenesis will be essential to fully understanding the roles of APOBEC enzymes in cancer etiology.

## Materials and methods

### RNA-seq and primary tumor mutation data sets

RSEM RNA-seq expression values for BRCA cell lines and TCGA sequenced primary tumors were obtained from the Cancer Cell Line Encyclopedia (https://data.broadinstitute.org/ccle/CCLE_RNAseq_rsem_transcripts_tpm_20180929.txt.gz) and the Broad Institute Genomic Data Analysis Center (http://gdac.broadinstitute.org/runs/stddata__2016_01_28/data/BRCA/20160128/gdac.broadinstitute.org_BRCA.Merge_rnaseqv2__illuminahiseq_rnaseqv2__unc_edu__Level_3__RSEM_genes_normalized__data.Level_3.2016012800.0.0.tar.gz; http://gdac.broadinstitute.org/runs/stddata__2016_01_28/data/BLCA/20160128/gdac.broadinstitute.org_BLCA.Merge_rnaseqv2__illuminahiseq_rnaseqv2__unc_edu__Level_3__RSEM_genes_normalized__data.Level_3.2016012800.0.0.tar.gz; http://gdac.broadinstitute.org/runs/stddata__2016_01_28/data/CESC/20160128/gdac.broadinstitute.org_CESC.Merge_rnaseqv2__illuminahiseq_rnaseqv2__unc_edu__Level_3__RSEM_genes__data.Level_3.2016012800.0.0.tar.gz; and http://gdac.broadinstitute.org/runs/stddata__2016_01_28/data/HNSC/20160128/gdac.broadinstitute.org_HNSC.Merge_rnaseqv2__illuminahiseq_rnaseqv2__unc_edu__Level_3__RSEM_genes_normalized__data.Level_3.2016012800.0.0.tar.gz), respectively. Raw RNA-seq reads for unambiguous RNA-seq analysis were obtained from the NCBI Short Read Archive for BRCA cell lines in the GEO series “Transcriptional profiling of a breast cancer cell line panel using RNAseq technology” (GEO accession number: GSE48213). Similar raw RNA-seq reads for TCGA sequenced primary BRCA tumors were downloaded as BAM Slices from the controlled-access portion of the NCI Genomics Data Commons (https://portal.gdc.cancer.gov/). Lists of mutations identified in BRCA cell lines were downloaded from the Cancer Cell Line Encyclopedia (https://data.broadinstitute.org/ccle/CCLE_DepMap_18Q1_maf_20180207.txt) and the COSMIC database (CosmicCLP_MutantExport.tsv.gz from https://cancer.sanger.ac.uk/cell_lines/download), while primary tumor mutations were obtained from http://gdac.broadinstitute.org/runs/stddata__2016_01_28/data/BRCA/20160128/gdac.broadinstitute.org_BRCA.Mutation_Packager_Oncotated_Calls.Level_3.2016012800.0.0.tar.gz; http://gdac.broadinstitute.org/runs/stddata__2016_01_28/data/BLCA/20160128/gdac.broadinstitute.org_BLCA.Mutation_Packager_Oncotated_Calls.Level_3.2016012800.0.0.tar.gz; http://gdac.broadinstitute.org/runs/stddata__2016_01_28/data/CESC/20160128/gdac.broadinstitute.org_CESC.Mutation_Packager_Oncotated_Calls.Level_3.2016012800.0.0.tar.gz; and http://gdac.broadinstitute.org/runs/stddata__2016_01_28/data/HNSC/20160128/gdac.broadinstitute.org_HNSC.Mutation_Packager_Oncotated_Calls.Level_3.2016012800.0.0.tar.gz.

### Cell culture

All breast cancer cell lines were obtained directly from ATCC as part of a breast cancer cell line panel, ATCC 30-4500K, and cultured utilizing conditions specified by ATCC. Antibiotic concentrations used for establishing stable cell populations are listed in (S4 Table). HEK293T cells were passaged in DMEM + 10% FBS at 5% CO2.

### shRNA mediated knockdown of APOBECs in BRCA cell lines

Oligonucleotides containing shRNA sequences targeting A3A, A3B, and a non-targeting scrambled sequence were cloned into pLKO.1 hygro (Addgene, #24150) to create lentiviral shRNA expression plasmids pTM-66 (A3A-shRNA), pTM-382 (A3B-shRNA-1), pTM-383 (A3B-shRNA-2), and pTM-238 (non-targeting shRNA). Plasmids psPAX2 (Addgene, #12260), pMD2.G (Addgene, #12259), and individual lentiviral shRNA plasmids were co-transfected into HEK293T cells for production of lentivirus. All the shRNA target sequences and oligonucleotides used for cloning are listed in S5 Table. Cell-free lentiviral supernatant was collected and used to transduce target cells in the presence of 8 μg/mL polybrene (MilliporeSigma) or Lentiblast transduction reagent (OZ biosciences), which was diluted 1:250 into lentiviral supernatant. Stable cell populations were selected with HygromycinB at concentrations listed in S4 Table. Although there are three mismatches between A3B-shRNA-1 and the A3A transcript-1 mRNA sequence, this shRNA, which is equivalent to Broad Institute TRCN0000140546, reduced A3A expression (S4 Fig). Therefore, pTM-383 (A3B-shRNA-2) was used for all A3B knockdown experiments.

### Generation of A3A CRISPR/Cas9 knockout lines

Oligonucleotides corresponding to five A3A-targeting gRNA sequences were cloned into the BsaI site of pGL3-U6-sgRNA-PGK-puromycin (Addgene, #51133) to create plasmids expressing sgRNAs targeting A3A. All the sgRNA target sequences and oligonucleotides used to create these plasmids are listed in the S5 Table. AU565 derived A3A knockout cell lines were generated by transiently co-transfecting AU565 cells with pST1374-nls-flag-cas9 (Addgene, #44758) and all five plasmids expressing A3A specific gRNAs. Cells expressing both Cas9 and gRNA were transiently selected by the addition of puromycin and blasticidin to the cell culture media for 3 days. Surviving cells were plated at approximately 100 cells / 10 cm dish and clonal cell lines were isolated via cloning cylinders. A3A knockouts were confirmed by PCR and sequencing using primers oTM-499 and oTM-501 (S2 Fig).

### Measurement of gene expression by qRT-PCR

RNA was isolated from cell populations in log-phase growth using homogenizer columns and a total RNA kit (Omega Bio-tek) and treated with DNAse I (ThermoFisher). cDNA was generated by combining 2 μg of denatured RNA from each sample with a reaction mixture (2.5 μM oligo dT_23_VN and 3.5 μM random hexamers, 0.5 mM dNTPs, 1x ProtoScript II Buffer, 10 mM DTT, 400U Protoscript II RT (NEB), and 16 U RNase Inhibitor (Superase-IN, Invitrogen) in a total volume of 40 μL. Reactions mixtures were incubated at 42°C for 1 hr, then 65°C for 20min and cDNA was stored at -20°C. qPCR was performed using 2 μL template with 1x SsoAdvanced Universal SYBR Green Supermix (Bio-Rad) and 0.2 μM primers (20 μL total reaction volume) on a Bio-Rad CFX96. Reactions were incubated at 95°C for 5 mins, then run for 40 cycles of 95°C for 10 s and 62.5°C for 1 min. Analysis of melt curves indicated singular products for all quantified products. Additionally, the qRT-PCR products were separated on an agarose gel to verify the products were of the expected sizes and lacked non-specific amplification (S14A Fig). The identities of qPCR products were validated by cloning products into plasmid pCR-Blunt (ThermoFisher) and sequencing 10 independent clones per qPCR reaction (S14B Fig). In order to minimize potentially confounding batch effects, the location of samples and primer sets was partially randomized using R x64 3.4.3. Primers for quantifying APOBEC transcripts are described in [36] and listed in the S5 Table.

### APOBEC3H haplotyping

cDNA was generated as described under the methods for qRT-PCR, except for the use of 5 μM oligo dT_23_VN as a primer. A3H was amplified utilizing KOD hot start DNA polymerase (MilliporeSigma) and a nested PCR approach. Products from 34 cycles of PCR with primers oTM-885 and oTM-883 were column purified and amplified for an additional 30 cycles with oTM-196 and oTM-884. The products were purified via PEG precipitation, cloned into pCR-Blunt, and Sanger sequenced with oTM-196 and oTM-884. Haplotypes were assigned as in [64].

### Mutation analysis in BRCA cell lines and TCGA tumors

Mutation lists for BRCA cell lines (from the Cancer Cell Line Encyclopedia and COSMIC) and TCGA-BRCA tumors (from the Broad GDAC) where subjected to statistical analyses similar to [1] to determine whether each sample was APOBEC-mutated, the total number of APOBEC- and non-APOBEC-induced mutations, and the minimum estimate of APOBEC-induced mutations. For the analysis of total mutation load in BRCA samples, the total number of APOBEC-induced mutations was determined as the number of TCW to TTW and TCW to TGW (including the complementary mutations WGA to WAA and WGA to WCA). Non-APOBEC-induced mutations were classified as all other mutations observed in the samples. APOBEC-mutated samples were defined as samples containing more APOBEC-induced mutations than expected by random mutagenesis. This was assessed statistically using a Fisher’s exact test comparing the number of APOBEC-induced mutations and non-APOBEC-induced mutations to the ratio of TCW to C bases (complementary sequences were combined) in a 40 bp window surrounding each mutation in a BRCA cell line from the COSMIC mutation list. Fisher’s exact p-values were adjusted for multiple hypothesis testing by Benjamini-Hochberg. BRCA cell lines with adjusted p-values of less than 0.05 with an odds ratio greater than 2 were classified as APOBEC-mutagenized; cell lines that did not meet these criteria were deemed non-APOBEC mutagenized. For comparisons of A3A and A3B expression to APOBEC-induced mutations, Log_10_ transformed qRT-PCR values, RSEM normalized RNA-seq values, or normalized unambiguous RNA-seq read values were compared to Log_10_ transformed values of the minimum estimate of APOBEC mutations. The minimum estimate of APOBEC-induced mutations in each sample was defined as the number of TCW to TTW and TCW to TGW (including the complementary mutations WGA to WAA and WGA to WCA) mutations in excess of what would be expected by random mutagenesis. Samples without a statistical over-representation of APOBEC-induced mutations display a minimum estimate of APOBEC-induced mutations equal to zero. Mutations in BRCA cell lines (obtained from the Cancer Cell Line Encyclopedia and COSMIC Cell Line Project) and TCGA-sequenced primary BRCA tumors were also de-convoluted into the COSMIC mutation signatures using the Mutational Patterns module in R [68]. The number of mutations occurring in Signatures 2 and 13 were totaled and Log_10_ transformed to compare to A3A and A3B expression.

### Lentiviral vectors for expression of UGI, APOBEC3A, and APOBEC3B

Plasmid pLenti CMV Puro DEST (Addgene, #17452) was modified in a series of cloning steps to create pTM-637, a lentiviral destination vector suitable for gateway cloning. pTM-637 has a modified CMV promoter for reduced expression and contains a pair of TETO sequences, which allows for doxycycline-induced expression in cells expressing TETR, and the CMV promoter and gateway module is orientated opposite the 5’ UTR. These attributes of pTM-637 prevent the expression of transgenes cloned into gateway module from being expressed in HEK-293T-TETR during lentiviral production, which was key for consistent production of high-titer lentivirus for the APOBEC expression vectors described below. Synthetic DNA fragments corresponding to the coding sequence (CDS) of A3A transcript 1 (NCBI accession NM_145699.4) with A3A intron 3, a C-terminal flexible linker and Strep-tag (A3A-geneblock) and the CDS of A3B transcript 1 (NCBI accession NM_004900.5) with A3B intron 6, a C-terminal flexible linker and Strep-tag (A3B-geneblock) were amplified with phosphorylated oligos oTM-58 and oTM-59, digested with EcoRV, and cloned into the HincII and EcoRV sites of pENTR1A no ccDB (Addgene, #17398) to produce plasmids pTM-216 and pTM-218, respectively. LR-Clonase (ThermoFisher) reactions between pTM-637 and pTM-216 or pTM-218 produced lentiviral vectors pTM-664 (A3A expression) and pTM-666 (A3B expression).

A synthetic DNA sequence encoding human-codon optimized Uracil Glycosylase inhibitor (UGI) from *Bacillus phage PBS2* was cloned into the EcoRV site of pENTR1A no ccDB to create pTM-775. A LR-Clonase reactions between pTM-775 and pLenti CMV Puro DEST or pLenti PGK Hygro DEST (Addgene, #19066) was used to create lentiviral vectors for UGI expression pTM-601 and pTM-553, respectively.

Sequences of synthetic DNA sequences used for A3A, A3B, and UGI cloning are provided in (S1 Text), GenBank formatted sequences for pTM-637, pTM-664, and pTM-666 will be made available from NCBI or Addgene (upon publication) and other sequences will be provided upon request.

### Whole-cell extracts

Whole-cell lysates were produced from approximately 5x10^6^ cells in log-phase growth. Prior to lysate generation, cells were washed twice with PBS, flash frozen in liquid nitrogen, and stored at -80°C. Lysates were generated by resuspending cells in 150 μL of lysis buffer (25mM HEPES, 150mM NaCl, 1mM EDTA, 10% Glycerol, 0.5% Triton X-100, and 2x Proteinase Inhibitor), and sonicating for 30 sec on setting “1” using a Misonix Sonicator 3000. Lysates were clarified by centrifugation at 13,000 g for 10 min at 4°C. Protein concentrations of lysates were measured by BCA assay and equal amounts of protein in lysates used for deaminase assays were confirmed by immunoblots as described below.

### Purification of APOBEC3A and APOBEC3B

The cell populations used for purification of A3A and A3B proteins were created by sequential transduction of HEK-293T cells with pLenti CMV TetR Blast (Addgene, #17492), pTM-553 (UGI), and either pTM-664 (A3A) or pTM-666 (A3B). Induction of A3A and A3B expression was achieved by seeding 1X10^6^ cells in 10 cm dishes in DMEM + 10% FBS + 2 μg/ml doxycycline. Cells were harvested at a density close to 9x10^6^ cells per 10 cm, which corresponded to approximately 84 hours of growth, washed twice with PBS, and frozen in liquid nitrogen.

Lysates for A3A and A3B purification were similarly prepared from cell pellets harvested from approximately 60- and 128–10 cm dishes, respectively. Cells lysates were prepared by addition of 30 mL of M-PER buffer (ThermoFisher) supplemented with 1 mM DTT and 2X Protease Inhibitor cocktail (SIGMAFAST, MilliporeSigma) and incubation at 4°C for 30 mins with gentle agitation. Lysates were subjected to three rounds of sonication on ice for 15 s on setting “5.5” using a Misonix Sonicator 3000. In order to reduce the nucleic acid content, MgCl_2_ and Benzonase (MilliporeSigma) were added to 1 mM and 40 U/mL respectively and lysates incubated at 4°C for 30 min. For A3B, RNAseA (Qiagen) was added to 6.6 μg/ml and the lysate was incubated 10 hours at 4°C, which was required to prevent RNA from co-purifying with A3B. To increase binding of the Strep-tagged protein and decrease non-specific protein binding during affinity purification, Tris-HCl pH 8, NaCl, and avidin were added to 15 mM, 50 mM, and 33 μg/mL respectively. Insoluble material was removed via centrifugation at 20,000 RCF for 30 mins and filtration through a 0.2 μM PES membrane.

A3A was purified as follows. A3A-Strep-tag was batch bound to 1 mL of Strep-Tactin Superflow Plus (Qiagen) at 4°C for 1 hr. The lysate was centrifuged at 400 RCF for 5 min at 4°C for and the supernatant removed from the Strep-Tactin resin. The Strep-Tactin resin was resuspended in 20 mL of buffer-1 (30 mM Tris-HCl (pH 7.9 at 25°C), 5% glycerol, 150 mM NaCl, 0.05% triton-100, 1 mM DTT) and transferred to a 1x20 cm glass econo-column. The Strep-Tactin resin was washed with an additional 40 mL of buffer-1 and A3A-strep-tag was eluted with buffer-1 containing 20 mM d-desthiobiotin. Fractions containing A3A-strep-tag were pooled and loaded to 1 mL Enrich-Q column (Bio-Rad) in buffer-1, washed with buffer-2 (30 mM Tris-HCl (pH 7.5 at 25°C), 5% glycerol, 150 mM NaCl, 0.05% triton-100, 1 mM DTT) and eluted utilizing a 20 mL linear gradient to 100% buffer-3 (30 mM Tris-HCl (pH 7.5 at 25°C), 5% glycerol, 1200 mM NaCl, 0.05% triton-100, 1 mM DTT). Purified A3A-strep-tag was extensively dialyzed in buffer-4 (30 mM Tris-HCl (pH 7.5 at 25°C), 10% glycerol, 150 mM NaCl, 0.05% triton-100, 1 mM DTT), frozen as aliquots in liquid nitrogen, and stored at -80°C.

A3B was purified as follows. A3B-Strep-tag containing lysate was loaded to a 1 mL Streptrap HP column (GE Healthcare) in buffer-5 (30 mM Tris-HCl (pH 7.9 at 25°C), 5% glycerol, 150 mM NaCl, 0.1% CHAPS, 1 mM DTT). The Streptrap HP column was then washed with 5 mL of buffer-5, 10 mL of buffer-6 (30 mM Tris-HCl (pH 7.9 at 25°C), 5% glycerol, 1000 mM NaCl, 0.1% CHAPS, 1 mM DTT) and 5 mL of buffer-7 (30 mM Tris-HCl (pH 7.9 at 25°C), 5% glycerol, 50 mM NaCl, 0.1% CHAPS, 1 mM DTT). A3B-strep-tag was eluted with buffer-7 containing 20 mM d-desthiobiotin. Approximately 6 mL of A3B-strep-tag containing fractions pooled and loaded to 1 mL Enrich-Q column (Bio-Rad) in buffer-1, washed with buffer-8 (30 mM Tris-HCl (pH 7.5 at 25°C), 5% glycerol, 50 mM NaCl, 0.1% CHAPS, 1 mM DTT) and eluted utilizing a 20 mL linear gradient to 100% buffer-9 (30 mM Tris-HCl (pH 7.5 at 25°C), 5% glycerol, 1200 mM NaCl, 0.1% CHAPS, 1 mM DTT) Purified A3B-strep-tag was extensively dialyzed in buffer-4 (30 mM Tris-HCl (pH 7.5 at 25°C), 10% glycerol, 150 mM NaCl, 0.05% CHAPS, 1 mM DTT), frozen as aliquots in liquid nitrogen, and stored at -80°C.

### Measurement of deaminase activity

Deaminase activity assays were performed with linear oligonucleotide oTM-910, /5Cy5/T*T* T*GTAGATGTAGATGTAGATTCAGTAGTAGAGTATGTAGT* A*T*T* T, and hairpin forming oligonucleotide substrates hairpin forming oligonucleotide substrates oTM-814, /5Cy5/TTTTATTTTGCAATTG**T**TCAATTGCAAAATTT*G*T*T. In S5 Fig, cytidine deaminase activity was assessed on both oTM-814 and oTM-818, /5Cy5/TTTTATTTTGCAATTG**A**TCAATTGCAAAATTT*G*T*T (the * denotes phosphorothioate bonds, which protect against nuclease degradation). These substrates contain a **Y**TCA, the A3A preferred motif or **R**TCA, the A3B preferred motif respectively. All other experiments assessing APOBEC cytidine deaminase activity on a hairpin substrate used only oTM-814. Deaminase activity assays with purified APOBEC enzymes (with concentrations A3A or A3B as indicated, 5 units of Uracil DNA glycosylase (NEB), 0.25 μM oligonucleotide substrate, 20 mM Tris HCl pH7.5, 1 mM DTT, and 1 mM EDTA), in a 20 μL volume were incubated for 30 minutes (unless otherwise indicated) at 37°C and terminated by the addition of 20 μL formamide buffer (47.5% formamide, 9mM EDTA, and 0.0125% SDS) and incubation at 95°C for 10 min. Deaminase activity assays with whole-cell lysates (40 μg of cell lysate, 0.25 μM oligonucleotide substrate, 22.5 mM Tris pH 7.56, 2 mM KCl, 2.5 mM NaCl, 0.005%Triton X-100, 1.5 mM DTT, and 1.25 mM EDTA), in a 20 μL volume were incubated for 24 hrs (unless otherwise indicated) at 37°C and terminated by the addition of 20 μL formamide buffer (47.5% formamide, 9 mM EDTA, and 0.0125% SDS). Reactions were incubated at 95°C for 10 min and placed on ice prior to electrophoresis. Deaminase activity assays were separated on pre-warmed 15% polyacrylamide gels with 7.9 M urea (7 cm) in 1x TBE buffer at 15 W for 15 min. Gels were imaged on a BioRad Chemidoc using the Cy5 setting. Percent substrate cleaved was determined by quantification of the intensity of the substrate and cytidine deamination cleavage product using BioRad’s Image Lab software version 5.2.

### RNA preparation from cytosolic and nuclear fractions

Nuclear and cytoplasmic RNA was prepared from 20–10 cm dishes of MDA-MB-453 cells. After harvesting the cells using trypsin, the cells were pelleted and washed twice with cold PBS. All subsequent steps were performed at 4°C. The cell pellet was resuspended in 10 mL of buffer CF (50 mM HEPES, 5 mM KCl, 140 mM NaCl, 1 mM EDTA, 0.8 M Hexylene Glycol, 1 mM DTT, 1X protease inhibitor cocktail) containing 25 μg/mL digitonin (MilliporeSigma) and incubated for 10 min with gentle agitation. This resulted in almost no cell lysis. The cells were pelleted by centrifugation at 2000 RCF for 10 minutes. The resulting cell pellet was resuspended in buffer CF supplemented with 0.5 mM NP-40 and incubated for 20 minutes with periodic gentle agitation. The nuclei were pelleted by centrifugation at 5000 RCF for 10 minutes. The supernatant (the cytoplasmic fraction) was removed, of which 1 mL was set aside for validation of fractionation by immunoblot. RNA was extracted from 7 mL of the cytoplasmic fraction by sequential phenol chloroform extraction, ethanol precipitation, and cleaned up using an RNA purification kit. The nuclei were washed 4 times in 40 mL of buffer CF. After the fourth wash, 10 percent of the total resuspended nuclei were transferred to a separate tube, pelleted, resuspended in buffer CF and lysed by sonication for validation of fractionation by immunoblot. The remaining nuclei were pelleted and used for RNA purification using an RNA purification kit.

### Immunoblots

40 μg of protein from each extract or subcellular fraction were separated on pre-made Mini Protean TGX gels (Bio-Rad) and run at 180 V for 35 min in 1xTGS buffer (25 mM Tris, 250 mM Glycine, 0.1% SDS). Gels were transferred via a Mini-Transblot Cell (Bio-Rad), at 55 V for 90 min at 4°C onto Immun-Blot PVDF membrane (BioRad) in 1xTransfer buffer (25 mM Tris, 192 mM Glycine, 20% (v/v) Methanol). Membranes were blocked in 2% ECL prime blocking reagent (GE Healthcare) in 1xTBST solution (20 mM Tris and 150 mM NaCl, 0.1% Tween-20). Anti-GAPDH (10494-I-AP, Proteintech), anti-TBP (8515S, Cell Signaling Technology), or anti-α-Tubulin (ab4074, Abcam) primary antibodies were used at 1:10000, 1:2000, and 1:20000 dilutions, respectively. A 1:20000 dilution of anti-rabbit secondary antibody (NA934-1ML, GE Healthcare) in 0.2% ECL Block in 1x TBST was used for all blots. Blots were developed with ECL Prime Western Blotting Detection Reagent (GE Healthcare) for 2 min before imaging on the BioRad Chemidoc MP Imaging System, using the Blots-Chemi setting.

### Unambiguous RNA-seq

To measure A3A and A3B expression in BRCA cell lines by RNA-seq without the complication of reads potentially mis-mapping between the highly homologous regions of the two genes, we conducted a pairwise alignment of the major Refseq transcripts for the *APOBEC3A* and *APOBEC3B* genes (NCBI accession numbers NM_145699.3 and NM_004900.4, respectively) with the Geneious Alignment module in Geneious v10.2.2 using Global alignment with free end gaps and a 93% similarity cost matrix. Regions in the 5’ and 3’ of each transcript that contain low sequence identity were identified. Raw RNA-seq reads were aligned with BWA using default settings to a reference genome containing the *APOBEC3A* and *APOBEC3B* transcripts (NCBI accession numbers NM_145699.3 and NM_004900.4, respectively). Reads aligning to regions between nucleotides 1 and 350 in the APOBEC3A transcript and displaying a CIGARSTRING of 75M, 101M or 105M (indicating complete matching of the read to the reference) were counted for each BRCA cell line, increased by 0.5, and compared to the total number of reads within the corresponding sequencing library. Similarly, reads mapping to the 1 to 622 and 1371 to 1560 regions of the APOBEC3B transcript and displaying a CIGARSTRINGs of 75M, 101M or 105M were counted, augmented by 0.5, and normalized to the total number of reads per sample. These values were Log_10_ transformed for analyses comparing A3A and A3B expression to minimum estimates of APOBEC-induced mutation in BRCA cell lines. Unambiguous RNA-seq for A3A and A3B expression in BRCA tumors was conducted by counting reads mapping to the genomic coordinates chr22:38957522–38957715 and chr22:38962652–38963183 for A3A transcripts and chr22:38982399–38986411 and chr22:38992611–38992779 for A3B transcripts, augmenting the counts by 0.5, and dividing the resulting values by the number of reads mapping to the *HPRT1* gene. As with unambiguous RNA-seq of BRCA cell lines, the normalized unambiguous RNA-seq values for BRCA tumors were Log_10_ transformed prior to statistical comparisons.

## Supporting information

S1 Text Sequences of synthetic DNA molecules used to construct APOBEC and UGI expression vectors. Key features include: EcoRV site used in cloning (bold green text), coding sequence (uppercase, bold blue text), intronic sequence (red text), AgeI sites that allow for exchanging epitope tags (yellow highlighted text), stop codon (uppercase, blue highlighting). (A) A3A-geneblock (B) A3B-geneblock (C) UGI-geneblock.
(DOCX)

S1 Fig A3A and A3B are more active on stem-loop substrates versus linear DNA substrates. Different amounts of purified A3A or A3B were incubated with either a linear DNA substrate (L) or hairpin substrate (H) containing YTCA deamination motifs for 3 hrs. Following addition of UDG to convert APOBEC-induced dU to a heat-liable abasic site, samples were incubated at 95°C and the cleavage products indicative of APOBEC activity (P) were resolved from the unreacted substrate (S) on a polyacrylamide denaturing gel.
(TIF)

S2 Fig Validation of CRISPR/Cas9 mediated deletion of APOBEC3A in AU565 cells. (A) Clonal cell lines were obtained following transfection of AU565 cells with Cas9 expression vector and APOBEC3A targeting guide RNAs. Genomic DNA was isolated from each line and the APOBEC3A gene amplified to identify lines with detectable disruptions in the gene following gel electrophoresis. Wild type APOBEC3A alleles produce an expected 715bp PCR product. CRISPR/Cas9 edited AU565 contains three disrupted APOBEC3A alleles. (B) Sanger Sequencing of the purified PCR products in the A3A deletion line. All three modified alleles generate either a premature stop codon or frameshift for A3A isoforms A and B.
(TIF)

S3 Fig Comparison of A3A and A3B expression to the number of COSMIC Signatures 2 and 13 mutations. The mutations utilized in Fig 2D and 2E were deconvoluted into COSMIC mutation signatures. The number of mutations in Signatures 2 and 13 (indicative of APOBEC-induced mutation) were summed and compared to the A3A and A3B mRNA transcript levels for 28 and 27 BRCA cell lines whose mutations were available from the Cancer Cell Line Encyclopedia and COSMIC Cell Line Project, respectively.
(TIF)

S4 Fig Specificity of shRNAs. A3B-shRNA-1 (equivalent to Broad Institute TRCN0000140546) reduced A3A mRNA expression in BT474 and AU565 derived cell populations. Newly derived A3A- and A3B-2-shRNAs are specific for their target genes and minimally impact expression of other APOBEC3 family members.
(TIF)

S5 Fig APOBEC3A is the predominant cytidine deaminase acting at RTCA motifs in BT474 cells. *In vitro* cytidine deaminase assay conducted as Fig 3D except using a hairpin substrate containing a RTCA target motif instead of a YTCA motif. Whole-cell extracts generated BT474 cells or BT474 cells transduced with lentiviral vectors to express scramble control, A3A-targeting, or A3B targeting shRNAs. Deaminase reactions were supplemented with either 2 units UGI or 50% glycerol added to the reaction.
(TIF)

S6 Fig Abundant APOBEC3A cytidine deaminase activity in CAMA-1 and MDA-MB-453 cells. (A) The mutation profile of CAMA-1 and MDA-MB-453 cells. (B) mRNA expression level of *APOBEC3A* and *APOBEC3B* relative to *HPRT1* measured by qRT-PCR in CAMA-1 or MDA-MB-453 cells and the corresponding cells transduced with lentiviral vectors to express scramble control, A3A-targeting, or A3B targeting shRNAs. CAMA-1 cells were also transduced with either vector-only control or UGI expression vectors. (C) *In vitro* cytidine deaminase assay (conducted similarly to Fig 1D and 1E) of whole-cell extracts generated from CAMA-1 or MDA-MB-453 cells in B. Deaminase reactions with MDA-MB-453 cells were supplemented with either 2 units UGI or and equal volume of 50% glycerol. Specificity of each shRNA was confirmed by qRT-PCR, and equal protein loading in deaminase assay verified by α-GAPDH western.
(TIF)

S7 Fig Correlation of cytidine deaminase activity with A3A and A3B mRNA expression level in untreated and RNAseA treated BRCA cell extracts. Whole cell extracts were generated from 10 BRCA cell lines (AU565, BT474, CAMA-1, HCC70, HCC202, MCF7, MDA-MB-361, MDA-MB-453, SKBR3, and T47D) and either untreated or treated with RNAseA to remove RNA from the extracts. These extracts were incubated with our hairpin oligonucleotide substrate containing an YTCA deamination target sequence for 24 hrs. Three independent assays were quantified and the resulting average activities were plotted against the average mRNA expression level of A3A and A3B measured by qRT-PCR. Error bars indicate the standard deviation in the cytidine deaminase activity measurements. Numerical values of the cytidine deaminase activity assays are provided in S6 Table.
(TIF)

S8 Fig *In vitro* A3A activity in the presence of high amounts of cellular RNA. 500 nM of A3A was incubated with 0.25 μM of hairpin DNA substrate containing an YTCA deamination target sequence for 30 minutes in the presence of 0, 100, and 2000 ng/μL RNA. Reactions were processed and quantified as in Fig 4.
(TIF)

S9 Fig A3B, but not A3A is inhibited by nuclear and cytoplasmic RNA. MDA-MB-453 cells were fractionated into nuclei and cytoplasm and RNA was isolated separately from each compartment. (A) Western blot analysis for the nuclear protein TBP and cytoplasmic protein tubulin confirming effective separation of the nuclei from cytoplasm. Increasing amounts of nuclear RNA (B) or cytoplasmic RNA (C) were added to *in vitro* cytidine deaminase assays containing 50 nM of either A3A or A3B and 0.25 μM of hairpin DNA substrate to assess the relative impact of RNA on A3A and A3B activity.
(TIF)

S10 Fig Alignment of the amino acid sequences for A3A and A3H. The blue arrows denote the five basic amino acids of A3H, which have been implicated in RNA binding. A3H-R175, R176 have been shown to be the major determinants of RNA-mediated inhibition of A3H activity [45]. A3A has a glycine at the residue corresponding to A3H-R175 and has only two basic amino acids in this region.
(TIF)

S11 Fig Similarity between RSEM normalized RNA-seq and unambiguous RNA-seq measurements of APOBEC3A and APOBEC3B expression. Comparison of RSEM normalized RNA-seq and unambiguous RNA-seq measurements of A3A and A3B expression among 23 BRCA cell lines or 1207 TCGA sequenced primary BRCA tumors was assessed by Pearson correlation test.
(TIF)

S12 Fig Comparison of A3A and A3B expression to the number of COSMIC Signatures 2 and 13 mutations in primary TCGA BRCA tumors. The mutations utilized in Fig 5 were deconvoluted into COSMIC mutation signatures. The number of mutations in Signatures 2 and 13 (indicative of APOBEC-induced mutation) were summed and compared to the A3A and A3B mRNA transcript levels for 577 primary BRCA tumors or 465 APOBEC-mutated BRCA tumors analyzed by the Mutational Patterns R package.
(TIF)

S13 Fig Correlation of A3A mRNA transcript level with the abundance of APOBEC-signature mutations in BRCA tumors expressing A3B at low levels. In primary BRCA tumors and the subset of APOBEC-mutated BRCA tumors, A3A expression positively correlates with A3B expression. However, in tumors restricted to the lowest quartile of A3B expression, the number of APOBEC-induced mutations still correlates with A3A mRNA transcript level. No correlation is observed between A3B expression and APOBEC mutagenesis in BRCA tumors expressing A3A at low levels.
(TIF)

S14 Fig Specificity of qRT-PCR products for APOBEC3 family members. (A) Endpoint qRT-PCR products for measuring the expression of individual APOBEC3 family members were separated based on size by agarose gel electrophoresis. All reactions produce a single product of the expected size for specific APOBEC3 family member assessed. (B) qRT-PCR products were cloned into the pCR-Blunt DNA vector and 9–10 independent clones were sequenced to verify the products were on-target. All sequences aligned to the target amplification region.
(TIF)

S1 Table Mutation characteristics of BRCA cell lines and primary tumors. (XLSX)

S2 Table APOBEC expression on BRCA cell lines and primary tumors. (XLSX)

S3 Table A3H Haplotypes of BRCA cell lines. (XLSX)

S4 Table Concentrations of antibiotics used to select transduced BRCA cells. (XLSX)

S5 Table Sequences of sgRNAs, shRNA targets, and oligonucleotides. (XLSX)

S6 Table Numerical values of cytidine deaminase activity assays in S7 Fig. (XLSX)

## References

[1] Roberts SA, Lawrence MS, Klimczak LJ, Grimm SA, Fargo D, Stojanov P, et al An APOBEC cytidine deaminase mutagenesis pattern is widespread in human cancers. Nat Genet. 2013;45(9):970–6. Epub 2013/07/16. 10.1038/ng.2702 23852170 PMC3789062

[2] Burns MB, Temiz NA, Harris RS. Evidence for APOBEC3B mutagenesis in multiple human cancers. Nat Genet. 2013;45(9):977–83. Epub 2013/07/16. 10.1038/ng.2701 23852168 PMC3902892

[3] Alexandrov LB, Nik-Zainal S, Wedge DC, Aparicio SA, Behjati S, Biankin AV, et al Signatures of mutational processes in human cancer. Nature. 2013;500(7463):415–21. Epub 2013/08/16. 10.1038/nature12477 23945592 PMC3776390

[4] Refsland EW, Harris RS. The APOBEC3 family of retroelement restriction factors. Curr Top Microbiol Immunol. 2013;371:1–27. Epub 2013/05/21. 10.1007/978-3-642-37765-5_1 23686230 PMC3934647

[5] Henderson S, Chakravarthy A, Su X, Boshoff C, Fenton TR. APOBEC-Mediated Cytosine Deamination Links PIK3CA Helical Domain Mutations to Human Papillomavirus-Driven Tumor Development. Cell Rep. 2014 Epub 2014/06/10. 10.1016/j.celrep.2014.05.012 . 24910434

[6] Roper N, Gao S, Maity TK, Banday AR, Zhang X, Venugopalan A, et al APOBEC Mutagenesis and Copy-Number Alterations Are Drivers of Proteogenomic Tumor Evolution and Heterogeneity in Metastatic Thoracic Tumors. Cell Rep. 2019;26(10):2651–66 e6. 10.1016/j.celrep.2019.02.028 . 30840888 PMC6461561

[7] Law EK, Sieuwerts AM, LaPara K, Leonard B, Starrett GJ, Molan AM, et al The DNA cytosine deaminase APOBEC3B promotes tamoxifen resistance in ER-positive breast cancer. Sci Adv. 2016;2(10):e1601737 10.1126/sciadv.1601737 27730215 PMC5055383

[8] Nikkila J, Kumar R, Campbell J, Brandsma I, Pemberton HN, Wallberg F, et al Elevated APOBEC3B expression drives a kataegic-like mutation signature and replication stress-related therapeutic vulnerabilities in p53-defective cells. Br J Cancer. 2017;117(1):113–23. 10.1038/bjc.2017.133 28535155 PMC5520199

[9] Green AM, Budagyan K, Hayer KE, Reed MA, Savani MR, Wertheim GB, et al Cytosine Deaminase APOBEC3A Sensitizes Leukemia Cells to Inhibition of the DNA Replication Checkpoint. Cancer Res. 2017;77(17):4579–88. 10.1158/0008-5472.CAN-16-3394 28655787 PMC5581702

[10] Buisson R, Lawrence MS, Benes CH, Zou L. APOBEC3A and APOBEC3B Activities Render Cancer Cells Susceptible to ATR Inhibition. Cancer Res. 2017;77(17):4567–78. 10.1158/0008-5472.CAN-16-3389 28698210 PMC5609510

[11] Roberts SA, Sterling J, Thompson C, Harris S, Mav D, Shah R, et al Clustered mutations in yeast and in human cancers can arise from damaged long single-strand DNA regions. Mol Cell. 2012;46(4):424–35. Epub 2012/05/23. 10.1016/j.molcel.2012.03.030 22607975 PMC3361558

[12] Nik-Zainal S, Alexandrov LB, Wedge DC, Van Loo P, Greenman CD, Raine K, et al Mutational processes molding the genomes of 21 breast cancers. Cell. 2012;149(5):979–93. 10.1016/j.cell.2012.04.024 22608084 PMC3414841

[13] Haradhvala NJ, Polak P, Stojanov P, Covington KR, Shinbrot E, Hess JM, et al Mutational Strand Asymmetries in Cancer Genomes Reveal Mechanisms of DNA Damage and Repair. Cell. 2016;164(3):538–49. 10.1016/j.cell.2015.12.050 26806129 PMC4753048

[14] Morganella S, Alexandrov LB, Glodzik D, Zou X, Davies H, Staaf J, et al The topography of mutational processes in breast cancer genomes. Nat Commun. 2016;7:11383 10.1038/ncomms11383 27136393 PMC5001788

[15] Seplyarskiy VB, Soldatov RA, Popadin KY, Antonarakis SE, Bazykin GA, Nikolaev SI. APOBEC-induced mutations in human cancers are strongly enriched on the lagging DNA strand during replication. Genome Res. 2016;26(2):174–82. 10.1101/gr.197046.115 26755635 PMC4728370

[16] Nik-Zainal S, Davies H, Staaf J, Ramakrishna M, Glodzik D, Zou X, et al Landscape of somatic mutations in 560 breast cancer whole-genome sequences. Nature. 2016;534(7605):47–54. 10.1038/nature17676 27135926 PMC4910866

[17] Bhagwat AS, Hao W, Townes JP, Lee H, Tang H, Foster PL. Strand-biased cytosine deamination at the replication fork causes cytosine to thymine mutations in Escherichia coli. Proc Natl Acad Sci U S A. 2016;113(8):2176–81. 10.1073/pnas.1522325113 26839411 PMC4776466

[18] Hoopes JI, Cortez LM, Mertz TM, Malc EP, Mieczkowski PA, Roberts SA. APOBEC3A and APOBEC3B Preferentially Deaminate the Lagging Strand Template during DNA Replication. Cell Rep. 2016;14(6):1273–82. 10.1016/j.celrep.2016.01.021 26832400 PMC4758883

[19] Saini N, Roberts SA, Sterling JF, Malc EP, Mieczkowski PA, Gordenin DA. APOBEC3B cytidine deaminase targets the non-transcribed strand of tRNA genes in yeast. DNA Repair (Amst). 2017;53:4–14. 10.1016/j.dnarep.2017.03.003 28351647 PMC5450012

[20] Green AM, Landry S, Budagyan K, Avgousti DC, Shalhout S, Bhagwat AS, et al APOBEC3A damages the cellular genome during DNA replication. Cell Cycle. 2016;15(7):998–1008. 10.1080/15384101.2016.1152426 26918916 PMC4889253

[21] Taylor BJ, Nik-Zainal S, Wu YL, Stebbings LA, Raine K, Campbell PJ, et al DNA deaminases induce break-associated mutation showers with implication of APOBEC3B and 3A in breast cancer kataegis. Elife. 2013;2:e00534 10.7554/eLife.00534 23599896 PMC3628087

[22] Burns MB, Lackey L, Carpenter MA, Rathore A, Land AM, Leonard B, et al APOBEC3B is an enzymatic source of mutation in breast cancer. Nature. 2013;494(7437):366–70. Epub 2013/02/08. 10.1038/nature11881 23389445 PMC3907282

[23] Starrett GJ, Luengas EM, McCann JL, Ebrahimi D, Temiz NA, Love RP, et al The DNA cytosine deaminase APOBEC3H haplotype I likely contributes to breast and lung cancer mutagenesis. Nat Commun. 2016;7:12918 10.1038/ncomms12918 27650891 PMC5036005

[24] Li MM, Emerman M. Polymorphism in human APOBEC3H affects a phenotype dominant for subcellular localization and antiviral activity. J Virol. 2011;85(16):8197–207. 10.1128/JVI.00624-11 21653666 PMC3147987

[25] Bogerd HP, Wiegand HL, Hulme AE, Garcia-Perez JL, O'Shea KS, Moran JV, et al Cellular inhibitors of long interspersed element 1 and Alu retrotransposition. Proc Natl Acad Sci U S A. 2006;103(23):8780–5. Epub 2006/05/27. 10.1073/pnas.0603313103 16728505 PMC1482655

[26] Chester A, Somasekaram A, Tzimina M, Jarmuz A, Gisbourne J, O'Keefe R, et al The apolipoprotein B mRNA editing complex performs a multifunctional cycle and suppresses nonsense-mediated decay. EMBO J. 2003;22(15):3971–82. 10.1093/emboj/cdg369 12881431 PMC169042

[27] Kidd JM, Newman TL, Tuzun E, Kaul R, Eichler EE. Population stratification of a common APOBEC gene deletion polymorphism. PLoS genetics. 2007;3(4):e63 10.1371/journal.pgen.0030063 17447845 PMC1853121

[28] Nik-Zainal S, Wedge DC, Alexandrov LB, Petljak M, Butler AP, Bolli N, et al Association of a germline copy number polymorphism of APOBEC3A and APOBEC3B with burden of putative APOBEC-dependent mutations in breast cancer. Nat Genet. 2014;46(5):487–91. 10.1038/ng.2955 24728294 PMC4137149

[29] Komatsu A, Nagasaki K, Fujimori M, Amano J, Miki Y. Identification of novel deletion polymorphisms in breast cancer. Int J Oncol. 2008;33(2):261–70. Epub 2008/07/19. . 18636146

[30] OhAinle M, Kerns JA, Li MM, Malik HS, Emerman M. Antiretroelement activity of APOBEC3H was lost twice in recent human evolution. Cell Host Microbe. 2008;4(3):249–59. 10.1016/j.chom.2008.07.005 18779051 PMC2608726

[31] Suspene R, Mussil B, Laude H, Caval V, Berry N, Bouzidi MS, et al Self-cytoplasmic DNA upregulates the mutator enzyme APOBEC3A leading to chromosomal DNA damage. Nucleic Acids Res. 2017;45(6):3231–41. 10.1093/nar/gkx001 28100701 PMC5389686

[32] Suspene R, Aynaud MM, Guetard D, Henry M, Eckhoff G, Marchio A, et al Somatic hypermutation of human mitochondrial and nuclear DNA by APOBEC3 cytidine deaminases, a pathway for DNA catabolism. Proc Natl Acad Sci U S A. 2011;108(12):4858–63. 10.1073/pnas.1009687108 21368204 PMC3064337

[33] Chan K, Roberts SA, Klimczak LJ, Sterling JF, Saini N, Malc EP, et al An APOBEC3A hypermutation signature is distinguishable from the signature of background mutagenesis by APOBEC3B in human cancers. Nat Genet. 2015;47(9):1067–72. 10.1038/ng.3378 26258849 PMC4594173

[34] Petljak M, Alexandrov LB, Brammeld JS, Price S, Wedge DC, Grossmann S, et al Characterizing Mutational Signatures in Human Cancer Cell Lines Reveals Episodic APOBEC Mutagenesis. Cell. 2019;176(6):1282–94 e20. 10.1016/j.cell.2019.02.012 . 30849372 PMC6424819

[35] Caval V, Suspene R, Shapira M, Vartanian JP, Wain-Hobson S. A prevalent cancer susceptibility APOBEC3A hybrid allele bearing APOBEC3B 3'UTR enhances chromosomal DNA damage. Nat Commun. 2014;5:5129 10.1038/ncomms6129 . 25298230

[36] Refsland EW, Stenglein MD, Shindo K, Albin JS, Brown WL, Harris RS. Quantitative profiling of the full APOBEC3 mRNA repertoire in lymphocytes and tissues: implications for HIV-1 restriction. Nucleic Acids Res. 2010;38(13):4274–84. 10.1093/nar/gkq174 20308164 PMC2910054

[37] Periyasamy M, Patel H, Lai CF, Nguyen VTM, Nevedomskaya E, Harrod A, et al APOBEC3B-Mediated Cytidine Deamination Is Required for Estrogen Receptor Action in Breast Cancer. Cell Rep. 2015;13(1):108–21. 10.1016/j.celrep.2015.08.066 26411678 PMC4597099

[38] Kouno T, Silvas TV, Hilbert BJ, Shandilya SMD, Bohn MF, Kelch BA, et al Crystal structure of APOBEC3A bound to single-stranded DNA reveals structural basis for cytidine deamination and specificity. Nat Commun. 2017;8:15024 10.1038/ncomms15024 28452355 PMC5414352

[39] Buisson R, Langenbucher A, Bowen D, Kwan EE, Benes CH, Zou L, et al Passenger hotspot mutations in cancer driven by APOBEC3A and mesoscale genomic features. Science. 2019;364(6447). 10.1126/science.aaw2872 . 31249028 PMC6731024

[40] Shi K, Carpenter MA, Banerjee S, Shaban NM, Kurahashi K, Salamango DJ, et al Structural basis for targeted DNA cytosine deamination and mutagenesis by APOBEC3A and APOBEC3B. Nat Struct Mol Biol. 2017;24(2):131–9. 10.1038/nsmb.3344 27991903 PMC5296220

[41] Di Noia J, Neuberger MS. Altering the pathway of immunoglobulin hypermutation by inhibiting uracil-DNA glycosylase. Nature. 2002;419(6902):43–8. 10.1038/nature00981 . 12214226

[42] Kanu N, Cerone MA, Goh G, Zalmas LP, Bartkova J, Dietzen M, et al DNA replication stress mediates APOBEC3 family mutagenesis in breast cancer. Genome Biol. 2016;17(1):185 10.1186/s13059-016-1042-9 27634334 PMC5025597

[43] Smith HC. RNA binding to APOBEC deaminases; Not simply a substrate for C to U editing. RNA Biol. 2017;14(9):1153–65. 10.1080/15476286.2016.1259783 27869537 PMC5699538

[44] Feng Y, Wong L, Morse M, Rouzina I, Williams MC, Chelico L. RNA-Mediated Dimerization of the Human Deoxycytidine Deaminase APOBEC3H Influences Enzyme Activity and Interaction with Nucleic Acids. J Mol Biol. 2018;430(24):4891–907. 10.1016/j.jmb.2018.11.006 30414963 PMC6289724

[45] Shaban NM, Shi K, Lauer KV, Carpenter MA, Richards CM, Salamango D, et al The Antiviral and Cancer Genomic DNA Deaminase APOBEC3H Is Regulated by an RNA-Mediated Dimerization Mechanism. Mol Cell. 2018;69(1):75–86 e9. 10.1016/j.molcel.2017.12.010 29290613 PMC5991973

[46] Bransteitter R, Pham P, Scharff MD, Goodman MF. Activation-induced cytidine deaminase deaminates deoxycytidine on single-stranded DNA but requires the action of RNase. Proc Natl Acad Sci U S A. 2003;100(7):4102–7. 10.1073/pnas.0730835100 12651944 PMC153055

[47] Chelico L, Pham P, Calabrese P, Goodman MF. APOBEC3G DNA deaminase acts processively 3' —> 5' on single-stranded DNA. Nat Struct Mol Biol. 2006;13(5):392–9. 10.1038/nsmb1086 . 16622407

[48] Xiao X, Yang H, Arutiunian V, Fang Y, Besse G, Morimoto C, et al Structural determinants of APOBEC3B non-catalytic domain for molecular assembly and catalytic regulation. Nucleic Acids Res. 2017;45(12):7540 10.1093/nar/gkx564 28645149 PMC5499557

[49] Ito F, Fu Y, Kao SA, Yang H, Chen XS. Family-Wide Comparative Analysis of Cytidine and Methylcytidine Deamination by Eleven Human APOBEC Proteins. J Mol Biol. 2017;429(12):1787–99. 10.1016/j.jmb.2017.04.021 28479091 PMC5530319

[50] Tang F, Lao K, Surani MA. Development and applications of single-cell transcriptome analysis. Nat Methods. 2011;8(4 Suppl):S6–11. 10.1038/nmeth.1557 21451510 PMC3408593

[51] Liu Z, Lee Y, Jang J, Li Y, Han X, Yokoi K, et al Microfluidic cytometric analysis of cancer cell transportability and invasiveness. Sci Rep. 2015;5:14272 10.1038/srep14272 26404901 PMC4585905

[52] Zetterberg A. Nuclear and cytoplasmic nucleic acid content and cytoplasmic protein synthesis during interphase in mouse fibroblasts in vitro. Exp Cell Res. 1966;43(3):517–25. 10.1016/0014-4827(66)90022-x . 5957742

[53] Land AM, Law EK, Carpenter MA, Lackey L, Brown WL, Harris RS. Endogenous APOBEC3A DNA cytosine deaminase is cytoplasmic and nongenotoxic. J Biol Chem. 2013;288(24):17253–60. 10.1074/jbc.M113.458661 23640892 PMC3682529

[54] Mussil B, Suspene R, Aynaud MM, Gauvrit A, Vartanian JP, Wain-Hobson S. Human APOBEC3A isoforms translocate to the nucleus and induce DNA double strand breaks leading to cell stress and death. PLoS One. 2013;8(8):e73641 10.1371/journal.pone.0073641 23977391 PMC3748023

[55] Landry S, Narvaiza I, Linfesty DC, Weitzman MD. APOBEC3A can activate the DNA damage response and cause cell-cycle arrest. EMBO Rep. 2011;12(5):444–50. 10.1038/embor.2011.46 21460793 PMC3090015

[56] Chen TW, Lee CC, Liu H, Wu CS, Pickering CR, Huang PJ, et al APOBEC3A is an oral cancer prognostic biomarker in Taiwanese carriers of an APOBEC deletion polymorphism. Nat Commun. 2017;8(1):465 10.1038/s41467-017-00493-9 28878238 PMC5587710

[57] Middlebrooks CD, Banday AR, Matsuda K, Udquim KI, Onabajo OO, Paquin A, et al Association of germline variants in the APOBEC3 region with cancer risk and enrichment with APOBEC-signature mutations in tumors. Nat Genet. 2016;48(11):1330–8. 10.1038/ng.3670 . 27643540 PMC6583788

[58] Waszak SM, Tiao G, Zhu B, Rausch T, Muyas F, Rodriguez-Martin B, et al Germline determinants of the somatic mutation landscape in 2,642 cancer genomes. bioRxiv. 2017:208330 10.1101/208330

[59] Maciejowski J, Chatzipli A, Dananberg A, de Lange T, Campbell PJ. APOBEC3B-dependent kataegis and TREX1-driven chromothripsis in telomere crisis. bioRxiv. 2019:725366 10.1101/725366 PMC7484228 32719516

[60] Basu U, Meng FL, Keim C, Grinstein V, Pefanis E, Eccleston J, et al The RNA exosome targets the AID cytidine deaminase to both strands of transcribed duplex DNA substrates. Cell. 2011;144(3):353–63. 10.1016/j.cell.2011.01.001 21255825 PMC3065114

[61] Pefanis E, Basu U. RNA Exosome Regulates AID DNA Mutator Activity in the B Cell Genome. Adv Immunol. 2015;127:257–308. 10.1016/bs.ai.2015.04.002 26073986 PMC4478610

[62] Soros VB, Yonemoto W, Greene WC. Newly synthesized APOBEC3G is incorporated into HIV virions, inhibited by HIV RNA, and subsequently activated by RNase H. PLoS Pathog. 2007;3(2):e15 10.1371/journal.ppat.0030015 17291161 PMC1796622

[63] Wang X, Abudu A, Son S, Dang Y, Venta PJ, Zheng YH. Analysis of human APOBEC3H haplotypes and anti-human immunodeficiency virus type 1 activity. J Virol. 2011;85(7):3142–52. 10.1128/JVI.02049-10 21270145 PMC3067873

[64] Ebrahimi D, Richards CM, Carpenter MA, Wang J, Ikeda T, Becker JT, et al Genetic and mechanistic basis for APOBEC3H alternative splicing, retrovirus restriction, and counteraction by HIV-1 protease. Nat Commun. 2018;9(1):4137 10.1038/s41467-018-06594-3 30297863 PMC6175962

[65] Matsumoto T, Shirakawa K, Yokoyama M, Fukuda H, Sarca AD, Koyabu S, et al Protein kinase A inhibits tumor mutator APOBEC3B through phosphorylation. Sci Rep. 2019;9(1):8307 10.1038/s41598-019-44407-9 31165764 PMC6549188

[66] Aynaud MM, Suspene R, Vidalain PO, Mussil B, Guetard D, Tangy F, et al Human Tribbles 3 protects nuclear DNA from cytidine deamination by APOBEC3A. J Biol Chem. 2012;287(46):39182–92. 10.1074/jbc.M112.372722 22977230 PMC3493958

[67] Hoopes JI, Hughes AL, Hobson LA, Cortez LM, Brown AJ, Roberts SA. Avoidance of APOBEC3B-induced mutation by error-free lesion bypass. Nucleic Acids Res. 2017;45(9):5243–54. 10.1093/nar/gkx169 28334887 PMC5605239

[68] Blokzijl F, Janssen R, van Boxtel R, Cuppen E. MutationalPatterns: comprehensive genome-wide analysis of mutational processes. Genome Med. 2018;10(1):33 10.1186/s13073-018-0539-0 29695279 PMC5922316

