# Supplementary material for: APOBEC3A is a prominent cytidine deaminase in breast cancer
Source: PLoS Genet. 2019 Dec 16;15(12):e1008545. doi: 10.1371/journal.pgen.1008545 (PMC6936861; doi:10.1371/journal.pgen.1008545)
Supplement: S1 Text — Key features include: EcoRV site used in cloning (bold green text), coding sequence (uppercase, bold blue text), intronic sequence (red text), AgeI sites that allow for exchanging epitope tags (yellow highlighted text), stop codon (uppercase, blue highlighting). (A) A3A-geneblock (B) A3B-geneblock (C) UGI-geneblock. (DOCX) [file pgen.1008545.s001.docx]

**S1 Text:**

Sequences of synthetic DNA molecules used to construct APOBEC and UGI expression vectors. Key features include: EcoRV site used in cloning (bold green text), coding sequence (uppercase, bold blue text), intronic sequence (red text), AgeI sites that allow for exchanging epitope tags (yellow highlighted text), stop codon (uppercase, blue highlighting). (A) A3A-geneblock (B) A3B-geneblock (C) UGI-geneblock

**(A) A3A-geneblock**

1 aattcaggtt ctgggagagg gta**gatatcA TGGAAGCCAG CCCAGCATCC GGGCCCAGAC**

**61 ACTTGATGGA TCCACACATA TTCACTTCCA ACTTTAACAA TGGCATTGGA AGGCATAAGA**

**121 CCTACCTGTG CTACGAAGTG GAGCGCCTGG ACAATGGCAC CTCGGTCAAG ATGGACCAGC**

**181 ACAGGGGCTT TCTACACAAC CAGGCTAAGA ATCTTCTCTG TGGCTTTTAC GGCCGCCATG**

**241 CGGAGCTGCG CTTCTTGGAC CTGGTTCCTT CTTTGCAGTT GGACCCGGCC CAGATCTACA**

**301 GGGTCACTTG GTTCATCTCC TGGAGCCCCT GCTTCTCCTG GGGCTGTGCC GGGGAAGTGC**

**361 GTGCGTTCCT TCAGGAGAAC ACACACGTGA GACTGCGTAT CTTCGCTGCC CGCATCTATG**

**421 ATTACGACCC CCTATATAAG GAGGCACTGC AAATGCTGCG GGATGCTGGG GCCCAAGTCT**

**481** **CCATCATGAC CTACGATG**gt aagaatggaa ggttcaggtg gggtggggtg ggtgggggca

541 ggagaggttc ctgggaagaa aaggagaaag gccttggtct gctgcctgca gaaacgatgg

601 ctggactctg ggacctgact ttggggtcga tgggaagaga gaggccaggc caggagatgt

661 gggcccaggg agggcaggga gagtggctgg aagtggaagc agaacttggg gctttctgaa

721 agaatgagaa ctgggctggc ccagattcca atgggaagga actgcctgat gaaggagcta

781 agtccctagg ggagggagag ggaaaggagg gactgaaacc aggatgtggg aagtctgtcc

841 tgagagtcat gggccctagg tgccaccccg atcccacagc gggagcgtga cttatctccc

901 ctgtcccttt tcag**AATTTA** **AGCACTGCTG GGACACCTTT GTGGACCACC AGGGATGTCC**

**961 CTTCCAGCCC TGGGATGGAC TAGATGAGCA CAGCCAAGCC CTGAGTGGGA GGCTGCGGGC**

**1021 CATTCTCCAG AATCAGGGAA AC**accgg**TGG AGGAGGCGGA TCCGGAGGAG GCGGATCCAG**

**1081 TGCTTGGAGT CATCCACAAT TCGAAAAAGG TGGAGGTTCC TAG**accggtt agggtaccca

1141 caggagaacg gggtgt

**(B) A3B-geneblock**

1 aattcaggtt ctgggagagg gta**gatatcA TGAATCCACA GATCAGAAAT CCGATGGAGC**

**61 GGATGTATCG AGACACATTC TACGACAACT TTGAAAACGA ACCCATCCTC TATGGTCGGA**

**121 GCTACACTTG GCTGTGCTAT GAAGTGAAAA TAAAGAGGGG CCGCTCAAAT CTCCTTTGGG**

**181 ACACAGGGGT CTTTCGAGGC CAGGTGTATT TCAAGCCTCA GTACCACGCA GAAATGTGCT**

**241 TCCTCTCTTG GTTCTGTGGC AACCAGCTGC CTGCTTACAA GTGTTTCCAG ATCACCTGGT**

**301 TTGTATCCTG GACCCCCTGC CCGGACTGTG TGGCGAAGCT GGCCGAATTC CTGTCTGAGC**

**361 ACCCCAATGT CACCCTGACC ATCTCTGCCG CCCGCCTCTA CTACTACTGG GAAAGAGATT**

**421 ACCGAAGGGC GCTCTGCAGG CTGAGTCAGG CAGGAGCCCG CGTGACGATC ATGGACTATG**

**481 AAGAATTTGC ATACTGCTGG GAAAACTTTG TGTACAATGA AGGTCAGCAA TTCATGCCTT**

**541 GGTACAAATT CGATGAAAAT TATGCATTCC TGCACCGCAC GCTAAAGGAG ATTCTCAGAT**

**601 ACCTGATGGA TCCAGACACA TTCACTTTCA ACTTTAATAA TGACCCTTTG GTCCTTCGAC**

**661 GGCGCCAGAC CTACTTGTGC TATGAGGTGG AGCGCCTGGA CAATGGCACC TGGGTCCTGA**

**721 TGGACCAGCA CATGGGCTTT CTATGCAACG AGGCTAAGAA TCTTCTCTGT GGCTTTTACG**

**781 GCCGCCATGC GGAGCTGCGC TTCTTGGACC TGGTTCCTTC TTTGCAGTTG GACCCGGCCC**

**841 AGATCTACAG GGTCACTTGG TTCATCTCCT GGAGCCCCTG CTTCTCCTGG GGCTGTGCCG**

**901 GGGAAGTGCG TGCGTTCCTT CAGGAGAACA CACACGTGAG ACTGCGCATC TTCGCTGCCC**

**961 GCATCTATGA TTACGACCCC CTATATAAGG AGGCGCTGCA AATGCTGCGG GATGCTGGGG**

**1021 CCCAAGTCTC CATCATGACC TACGATG**gta agaatggaag gttcaggtgg ggtggggtgg

1081 gtgggggcag gagaggttcc tgggaagaaa aggagaaagg ccttggtctg ctgcctgcag

1141 aaacgatggc tggactctgg gacctgactt tggggtcgat gggaagagag aggccaggcc

1201 aggagatatg ggcccgggga gggtggctgg aagtggaagc agaacttggg gatttccgaa

1261 agaaagagaa ctgggctggc ccagattcca atgggaagga agtacctgat gaaggagcta

1321 agtccctagg ggagggagag ggaaaggagg gactgaaacc aggatgtggg aagtctgtcc

1381 tgagagtcat gggcccttgg tgctgccccc tccccacaac aggagcgtga cttatctccc

1441 ctgtcccttt tcag**AGTTTG** **AGTACTGCTG GGACACCTTT GTGTACCGCC AGGGATGTCC**

**1501 CTTCCAGCCC TGGGATGGAC TAGAGGAGCA CAGCCAAGCC CTGAGTGGGA GGCTGCGGGC**

**1561 CATTCTCCAG AATCAGGGAA AC**accggt**GG** **AGGAGGCGGA TCCGGAGGAG GCGGATCCAG**

**1621 TGCTTGGAGT CATCCACAAT TCGAAAAAGG TGGAGGTTCC TAG**accggtt agggtaccca

1681 caggagaacg gggtgt

**(C) UGI-geneblock**

**1 ATGACCAACC TGAGCGACAT CATCGAGAAG GAGACCGGCA AGCAGCTGGT GATCCAGGAG**

**61 AGCATCCTGA TGCTGCCCGA GGAGGTGGAG GAGGTGATCG GCAACAAGCC CGAGAGCGAC**

**121 ATCCTGGTGC ACACCGCCTA CGACGAGAGC ACCGACGAGA ACGTGATGCT GCTGACCAGC**

**181 GACGCCCCCG AGTACAAGCC CTGGGCCCTG GTGATCCAGG ACAGCAACGG CGAGAACAAG**

**241 ATCAAGATGC TG**
