# Supplementary figures and images for: APOBEC3A is a prominent cytidine deaminase in breast cancer
Source: PLoS Genet. 2019 Dec 16;15(12):e1008545. doi: 10.1371/journal.pgen.1008545 (PMC6936861; doi:10.1371/journal.pgen.1008545)

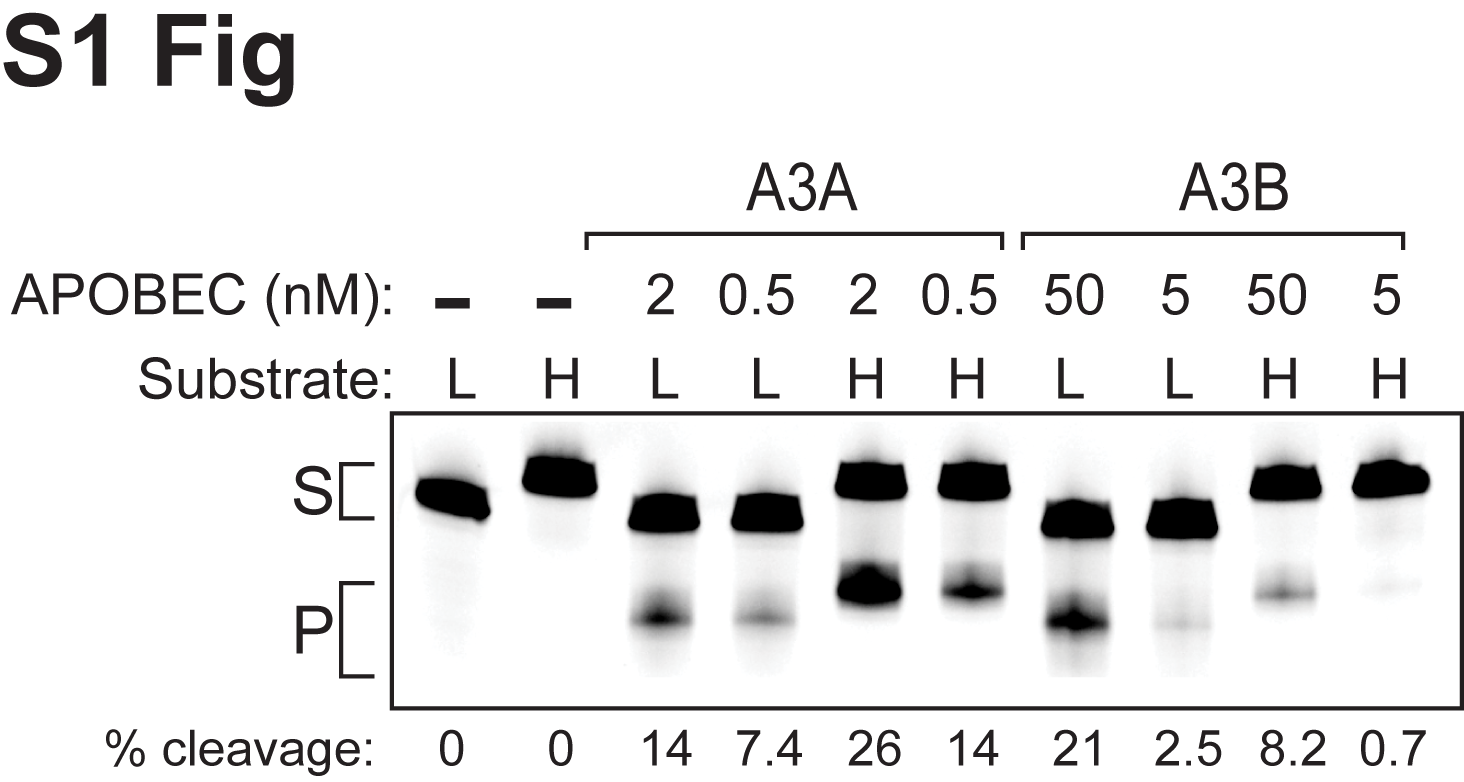

Supplement: S1 Fig — Different amounts of purified A3A or A3B were incubated with either a linear DNA substrate (L) or hairpin substrate (H) containing YTCA deamination motifs for 3 hrs. Following addition of UDG to convert APOBEC-induced dU to a heat-liable abasic site, samples were incubated at 95°C and the cleavage products indicative of APOBEC activity (P) were resolved from the unreacted substrate (S) on a polyacrylamide denaturing gel. (TIF) [file pgen.1008545.s002.tif]

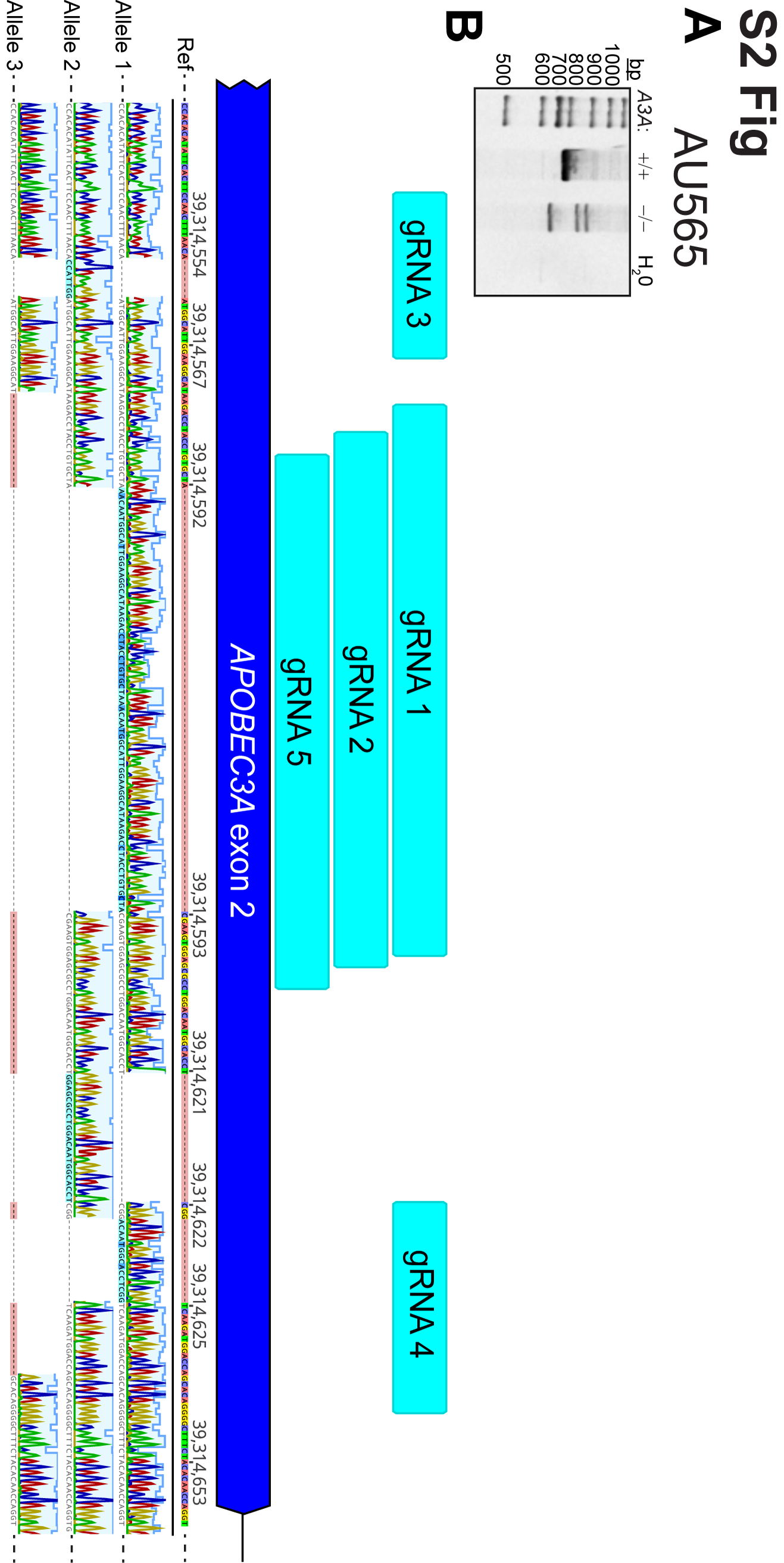

Supplement: S2 Fig — (A) Clonal cell lines were obtained following transfection of AU565 cells with Cas9 expression vector and APOBEC3A targeting guide RNAs. Genomic DNA was isolated from each line and the APOBEC3A gene amplified to identify lines with detectable disruptions in the gene following gel electrophoresis. Wild type APOBEC3A alleles produce an expected 715bp PCR product. CRISPR/Cas9 edited AU565 contains three disrupted APOBEC3A alleles. (B) Sanger Sequencing of the purified PCR products in the A3A deletion line. All three modified alleles generate either a premature stop codon or frameshift for A3A isoforms A and B. (TIF) [file pgen.1008545.s003.tif]

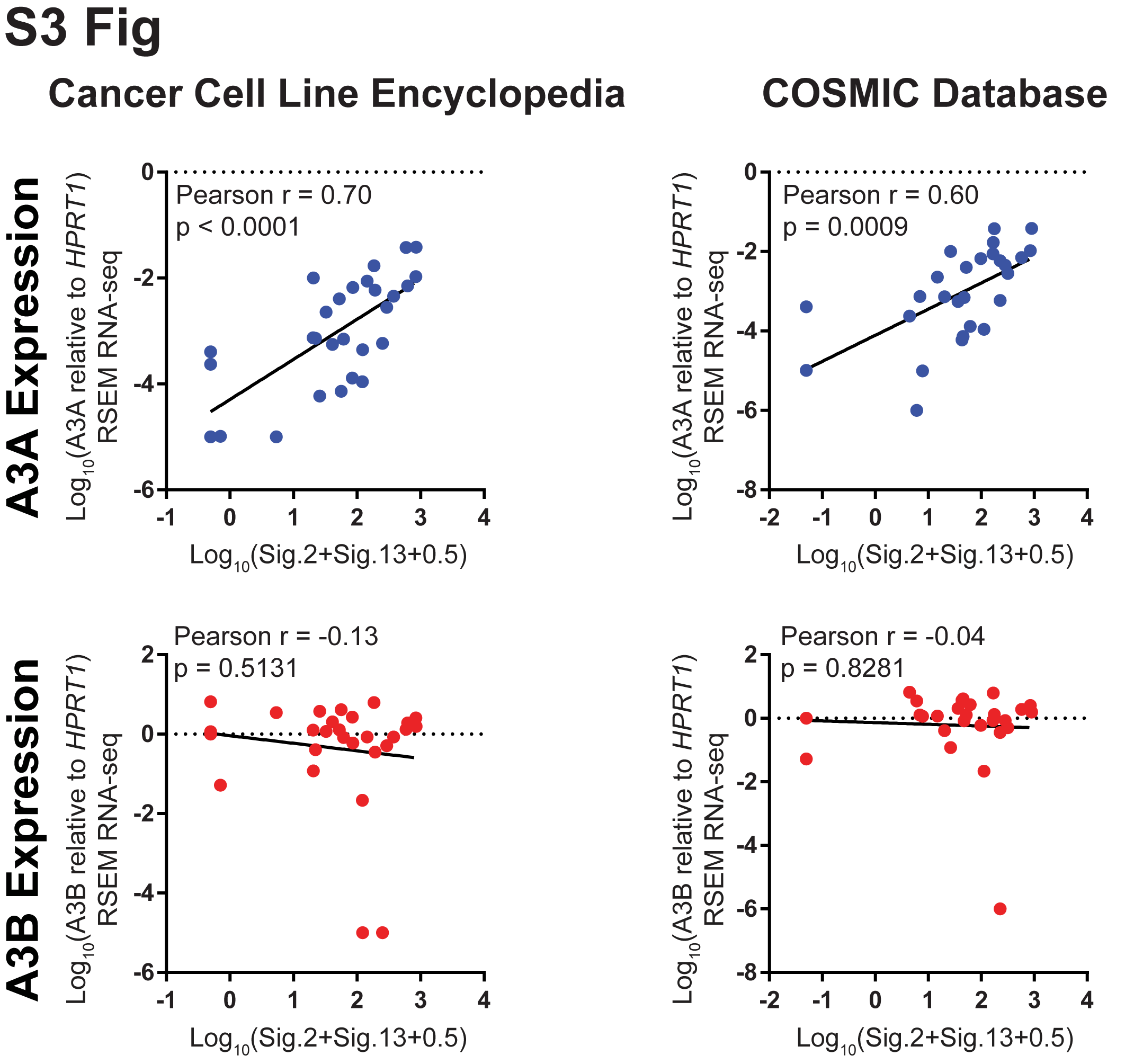

Supplement: S3 Fig — The mutations utilized in Fig 2D and 2E were deconvoluted into COSMIC mutation signatures. The number of mutations in Signatures 2 and 13 (indicative of APOBEC-induced mutation) were summed and compared to the A3A and A3B mRNA transcript levels for 28 and 27 BRCA cell lines whose mutations were available from the Cancer Cell Line Encyclopedia and COSMIC Cell Line Project, respectively. (TIF) [file pgen.1008545.s004.tif]

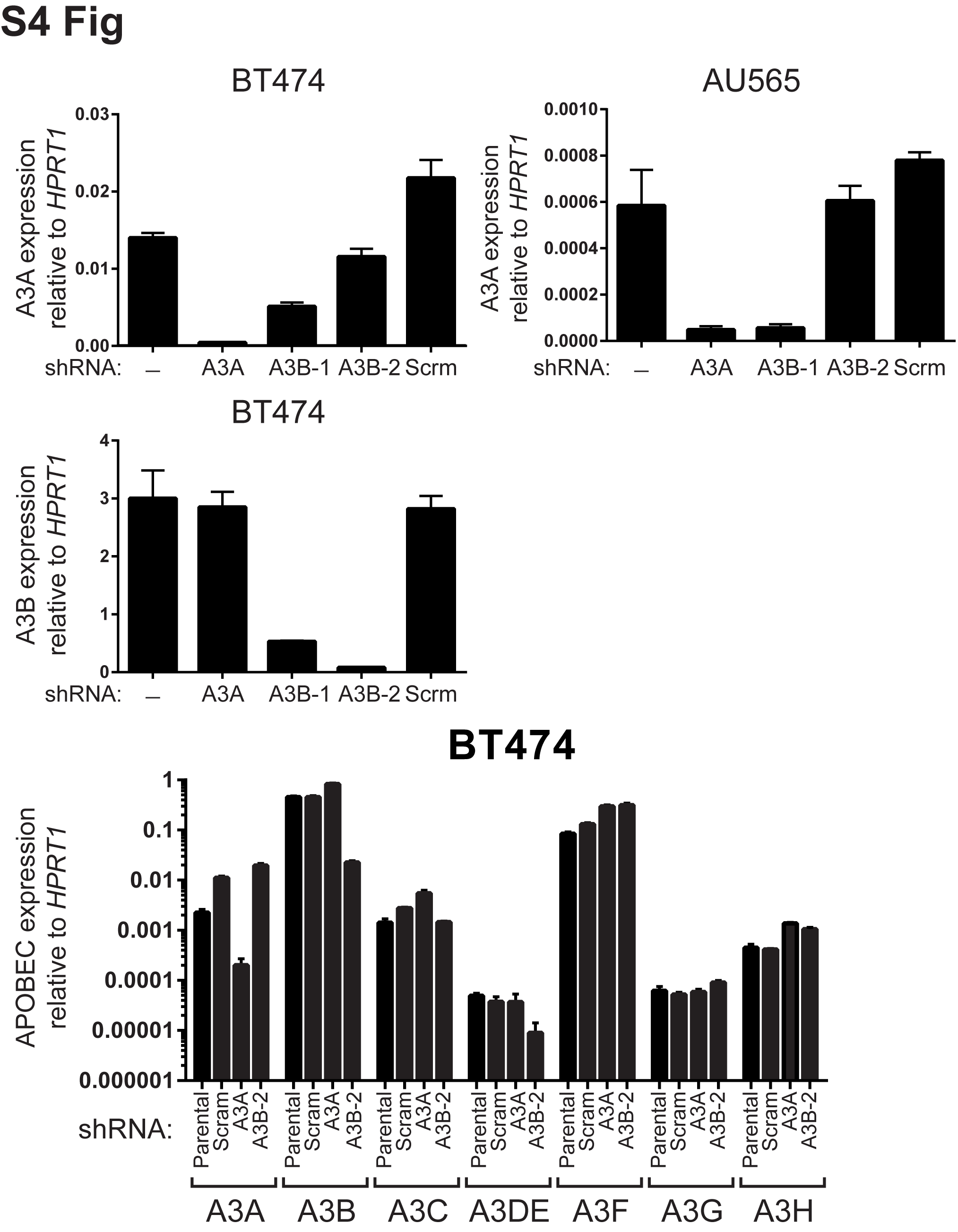

Supplement: S4 Fig — A3B-shRNA-1 (equivalent to Broad Institute TRCN0000140546) reduced A3A mRNA expression in BT474 and AU565 derived cell populations. Newly derived A3A- and A3B-2-shRNAs are specific for their target genes and minimally impact expression of other APOBEC3 family members. (TIF) [file pgen.1008545.s005.tif]

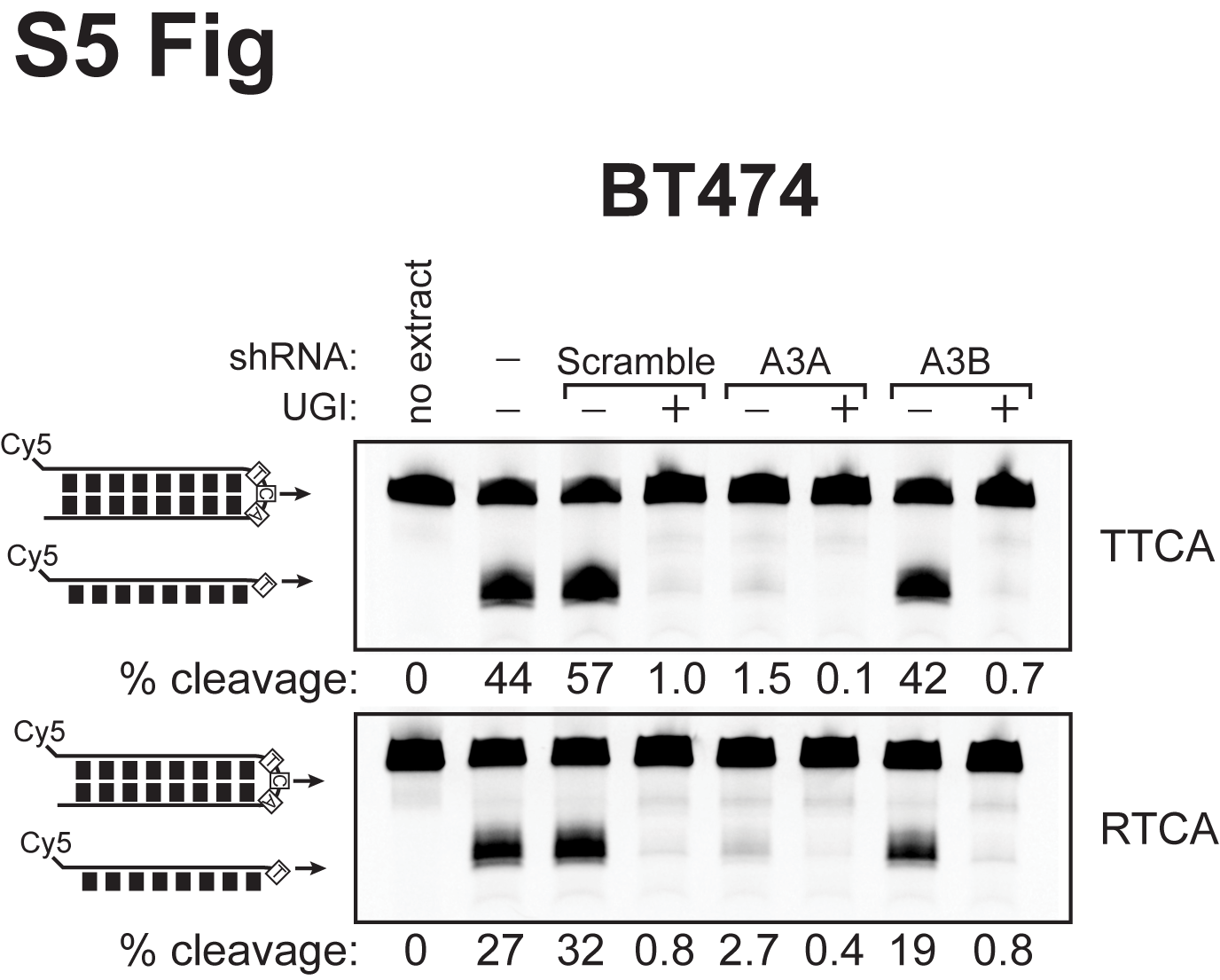

Supplement: S5 Fig — In vitro cytidine deaminase assay conducted as Fig 3D except using a hairpin substrate containing a RTCA target motif instead of a YTCA motif. Whole-cell extracts generated BT474 cells or BT474 cells transduced with lentiviral vectors to express scramble control, A3A-targeting, or A3B targeting shRNAs. Deaminase reactions were supplemented with either 2 units UGI or 50% glycerol added to the reaction. (TIF) [file pgen.1008545.s006.tif]

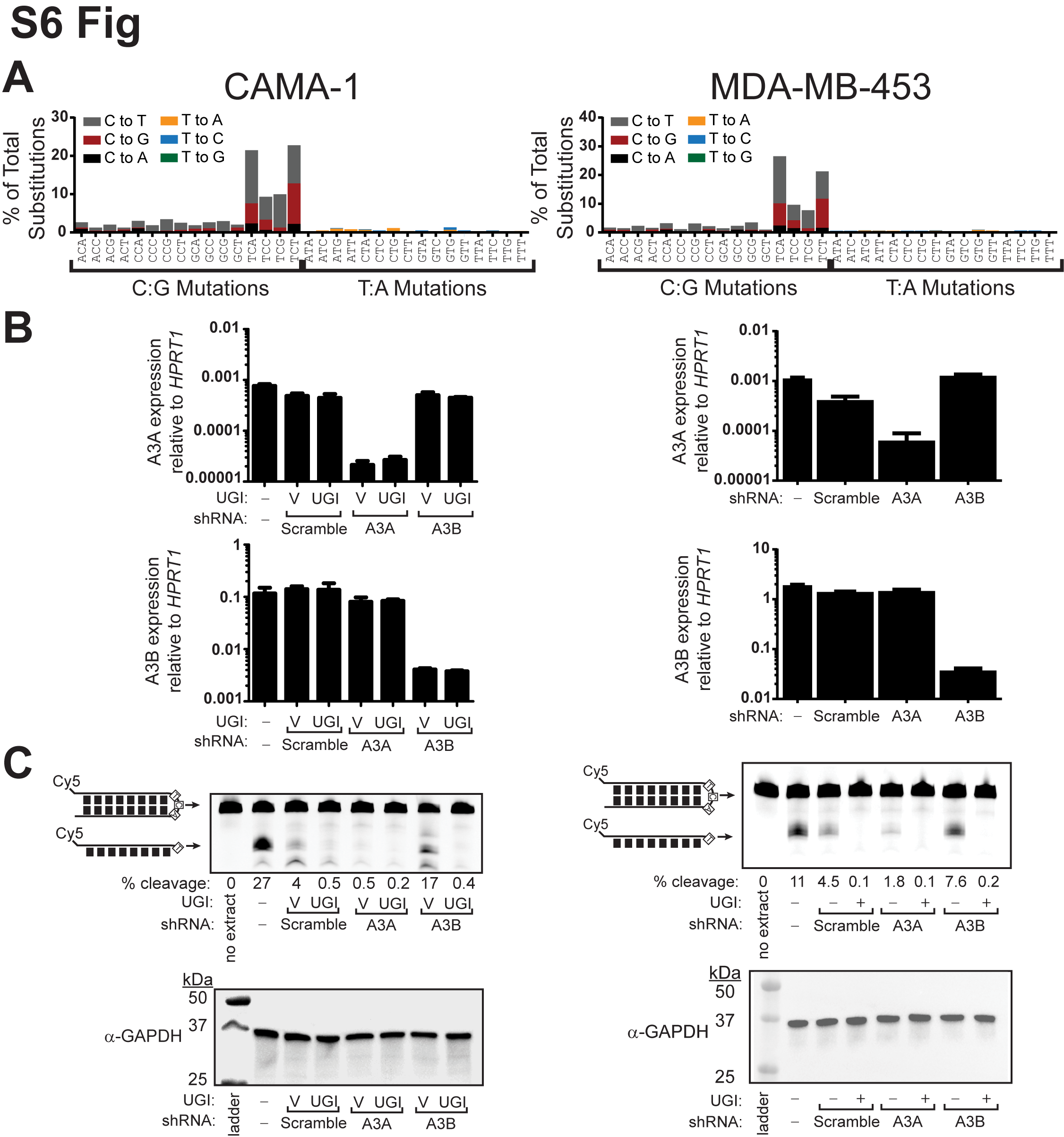

Supplement: S6 Fig — (A) The mutation profile of CAMA-1 and MDA-MB-453 cells. (B) mRNA expression level of APOBEC3A and APOBEC3B relative to HPRT1 measured by qRT-PCR in CAMA-1 or MDA-MB-453 cells and the corresponding cells transduced with lentiviral vectors to express scramble control, A3A-targeting, or A3B targeting shRNAs. CAMA-1 cells were also transduced with either vector-only control or UGI expression vectors. (C) In vitro cytidine deaminase assay (conducted similarly to Fig 1D and 1E) of whole-cell extracts generated from CAMA-1 or MDA-MB-453 cells in B. Deaminase reactions with MDA-MB-453 cells were supplemented with either 2 units UGI or and equal volume of 50% glycerol. Specificity of each shRNA was confirmed by qRT-PCR, and equal protein loading in deaminase assay verified by α-GAPDH western. (TIF) [file pgen.1008545.s007.tif]

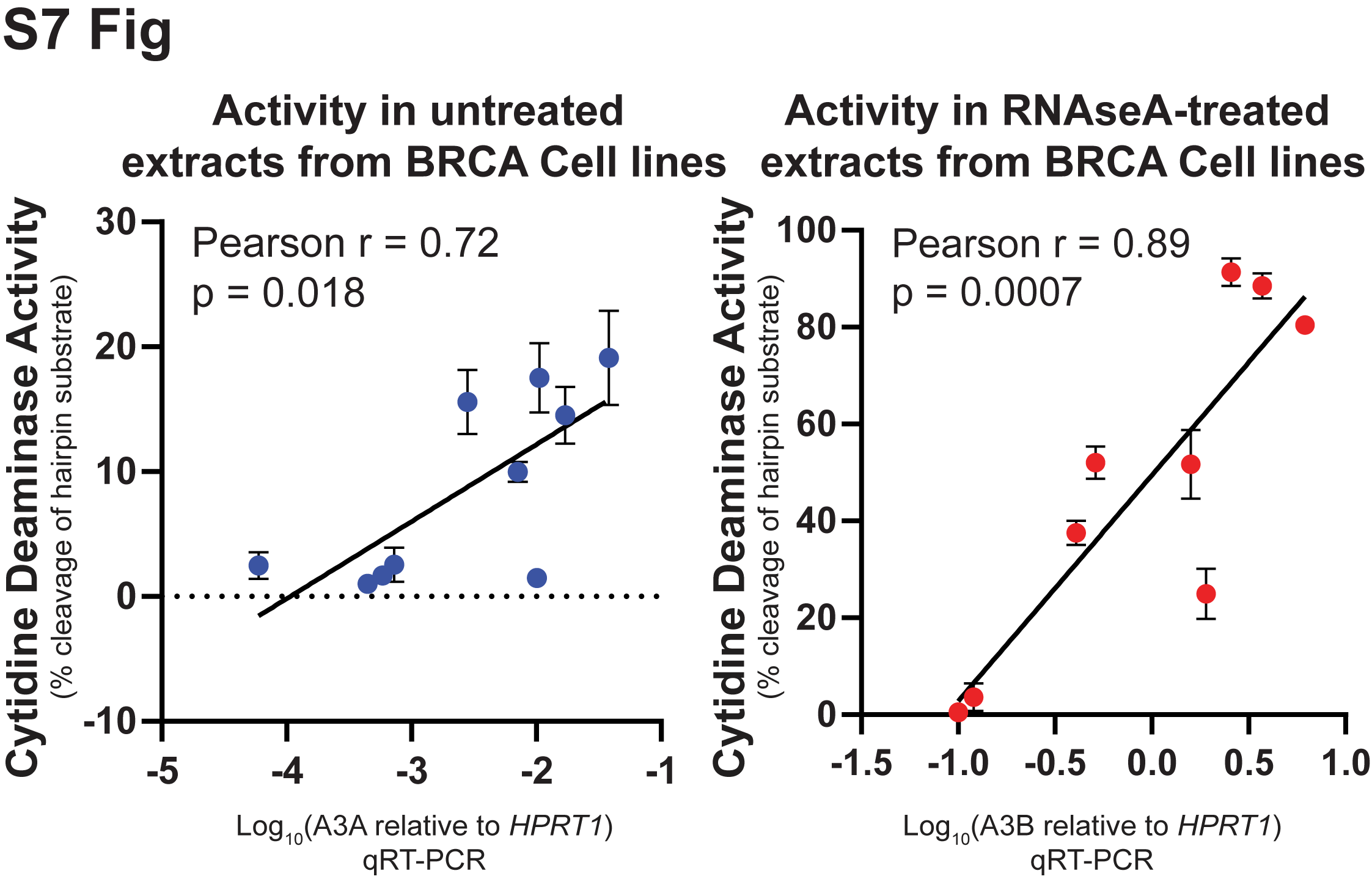

Supplement: S7 Fig — Whole cell extracts were generated from 10 BRCA cell lines (AU565, BT474, CAMA-1, HCC70, HCC202, MCF7, MDA-MB-361, MDA-MB-453, SKBR3, and T47D) and either untreated or treated with RNAseA to remove RNA from the extracts. These extracts were incubated with our hairpin oligonucleotide substrate containing an YTCA deamination target sequence for 24 hrs. Three independent assays were quantified and the resulting average activities were plotted against the average mRNA expression level of A3A and A3B measured by qRT-PCR. Error bars indicate the standard deviation in the cytidine deaminase activity measurements. Numerical values of the cytidine deaminase activity assays are provided in S6 Table. (TIF) [file pgen.1008545.s008.tif]

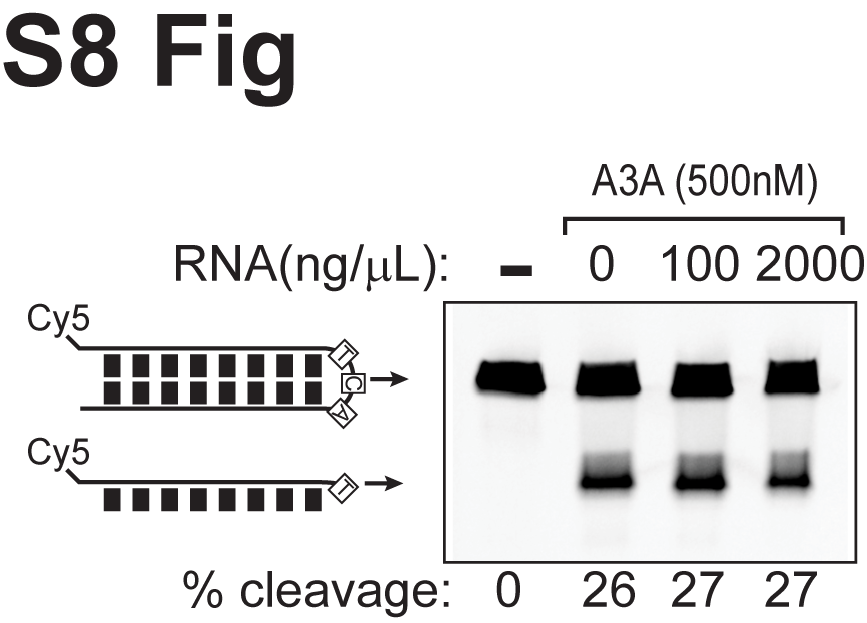

Supplement: S8 Fig — 500 nM of A3A was incubated with 0.25 μM of hairpin DNA substrate containing an YTCA deamination target sequence for 30 minutes in the presence of 0, 100, and 2000 ng/μL RNA. Reactions were processed and quantified as in Fig 4. (TIF) [file pgen.1008545.s009.tif]

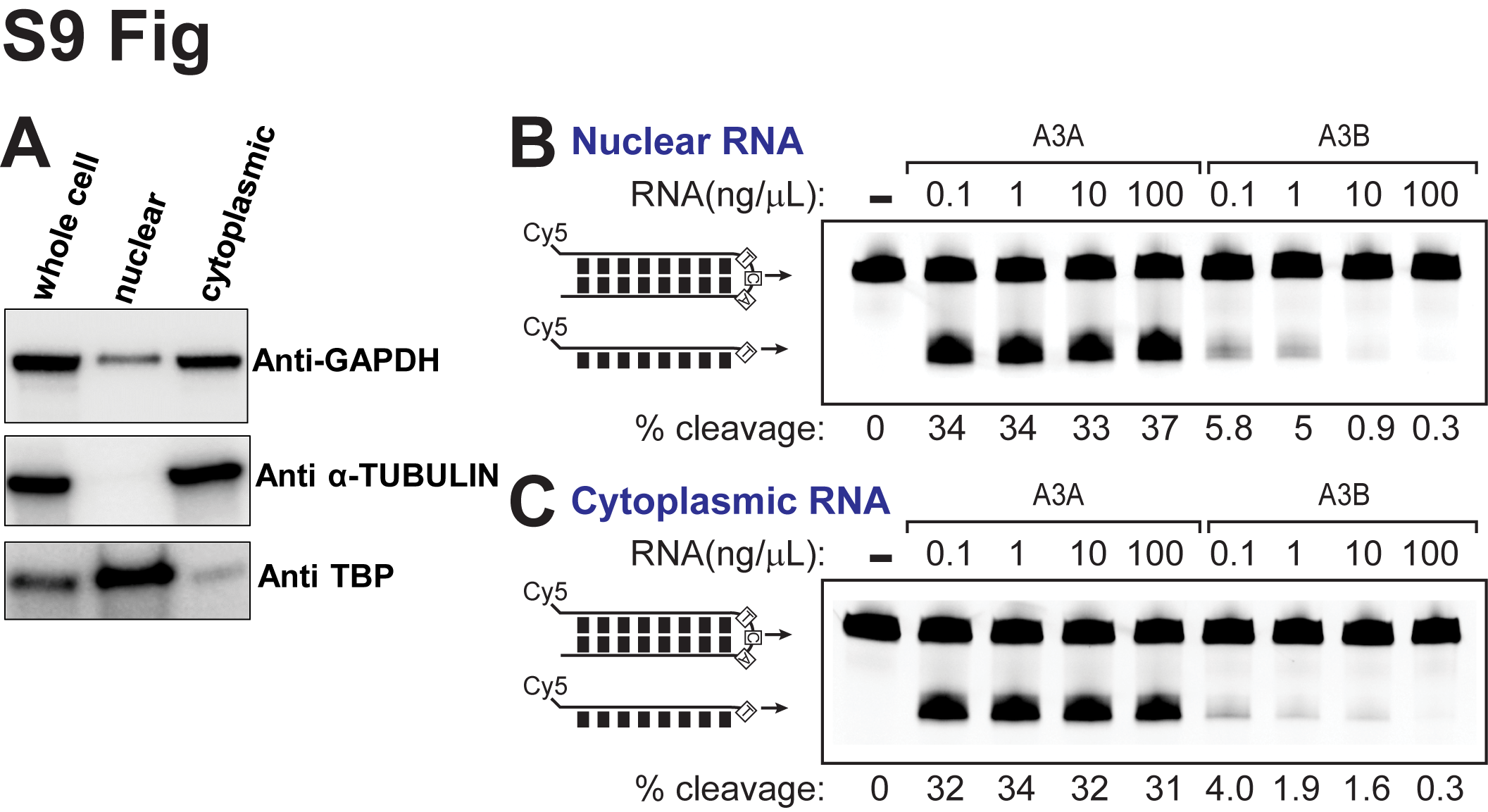

Supplement: S9 Fig — MDA-MB-453 cells were fractionated into nuclei and cytoplasm and RNA was isolated separately from each compartment. (A) Western blot analysis for the nuclear protein TBP and cytoplasmic protein tubulin confirming effective separation of the nuclei from cytoplasm. Increasing amounts of nuclear RNA (B) or cytoplasmic RNA (C) were added to in vitro cytidine deaminase assays containing 50 nM of either A3A or A3B and 0.25 μM of hairpin DNA substrate to assess the relative impact of RNA on A3A and A3B activity. (TIF) [file pgen.1008545.s010.tif]

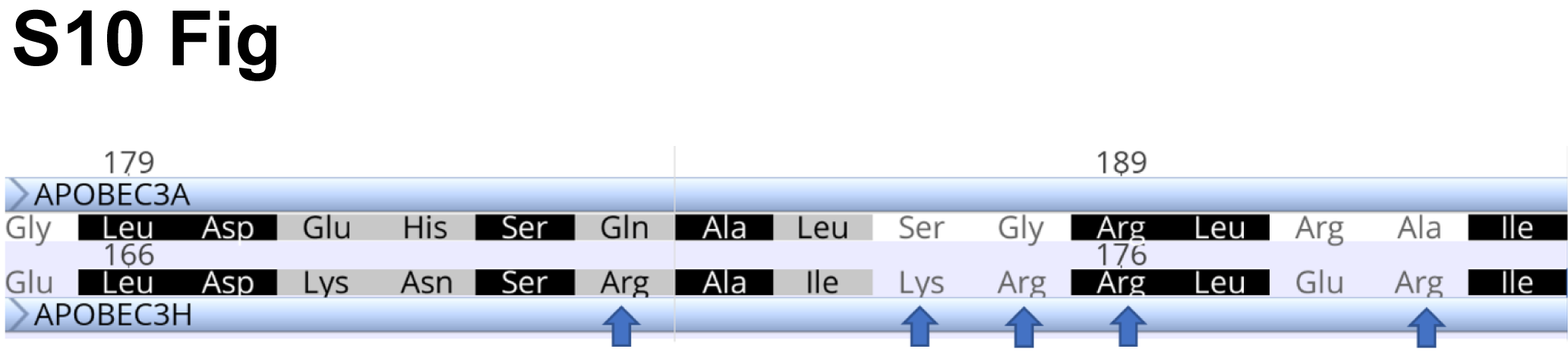

Supplement: S10 Fig — The blue arrows denote the five basic amino acids of A3H, which have been implicated in RNA binding. A3H-R175, R176 have been shown to be the major determinants of RNA-mediated inhibition of A3H activity [45]. A3A has a glycine at the residue corresponding to A3H-R175 and has only two basic amino acids in this region. (TIF) [file pgen.1008545.s011.tif]

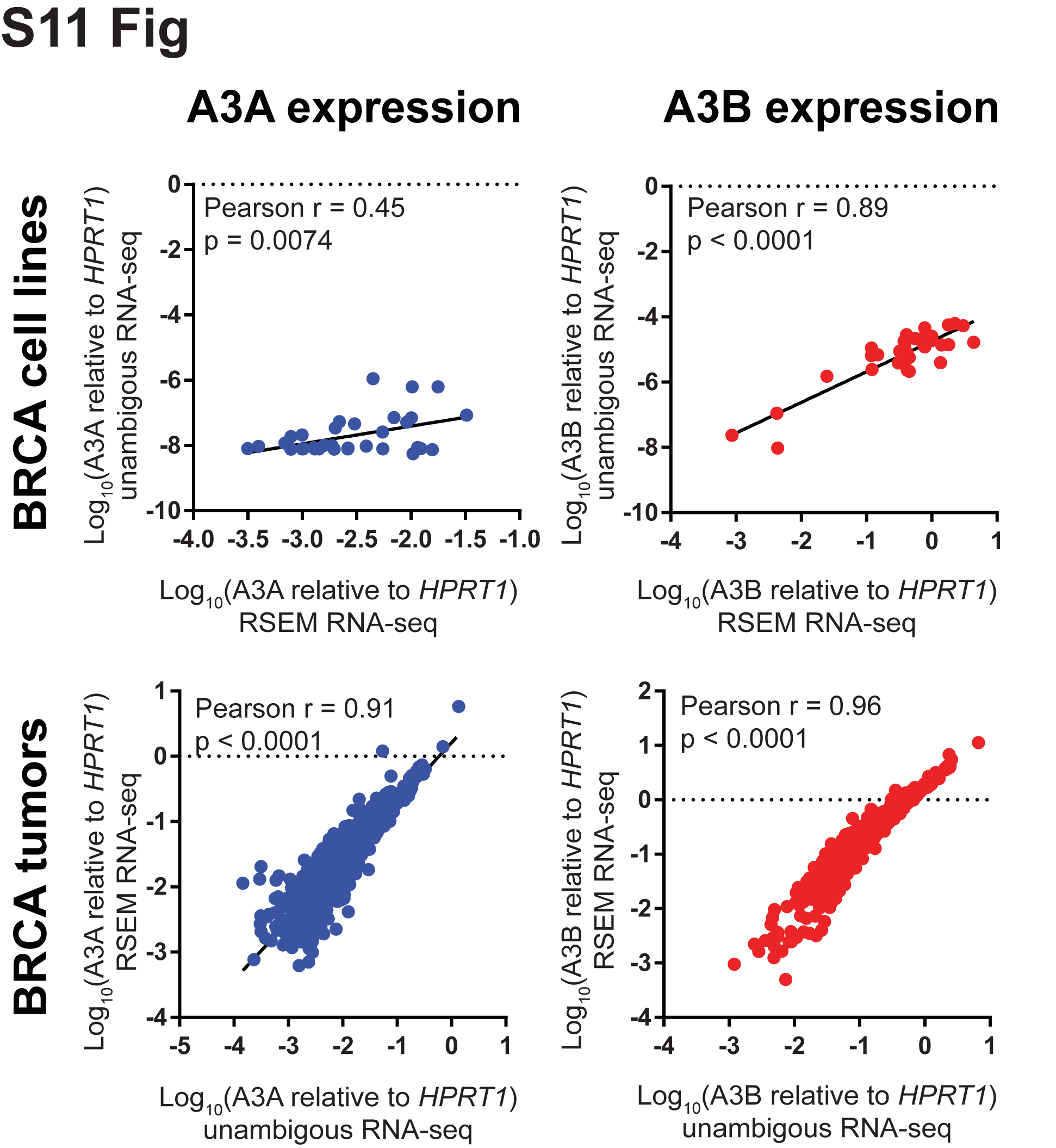

Supplement: S11 Fig — Comparison of RSEM normalized RNA-seq and unambiguous RNA-seq measurements of A3A and A3B expression among 23 BRCA cell lines or 1207 TCGA sequenced primary BRCA tumors was assessed by Pearson correlation test. (TIF) [file pgen.1008545.s012.tif]

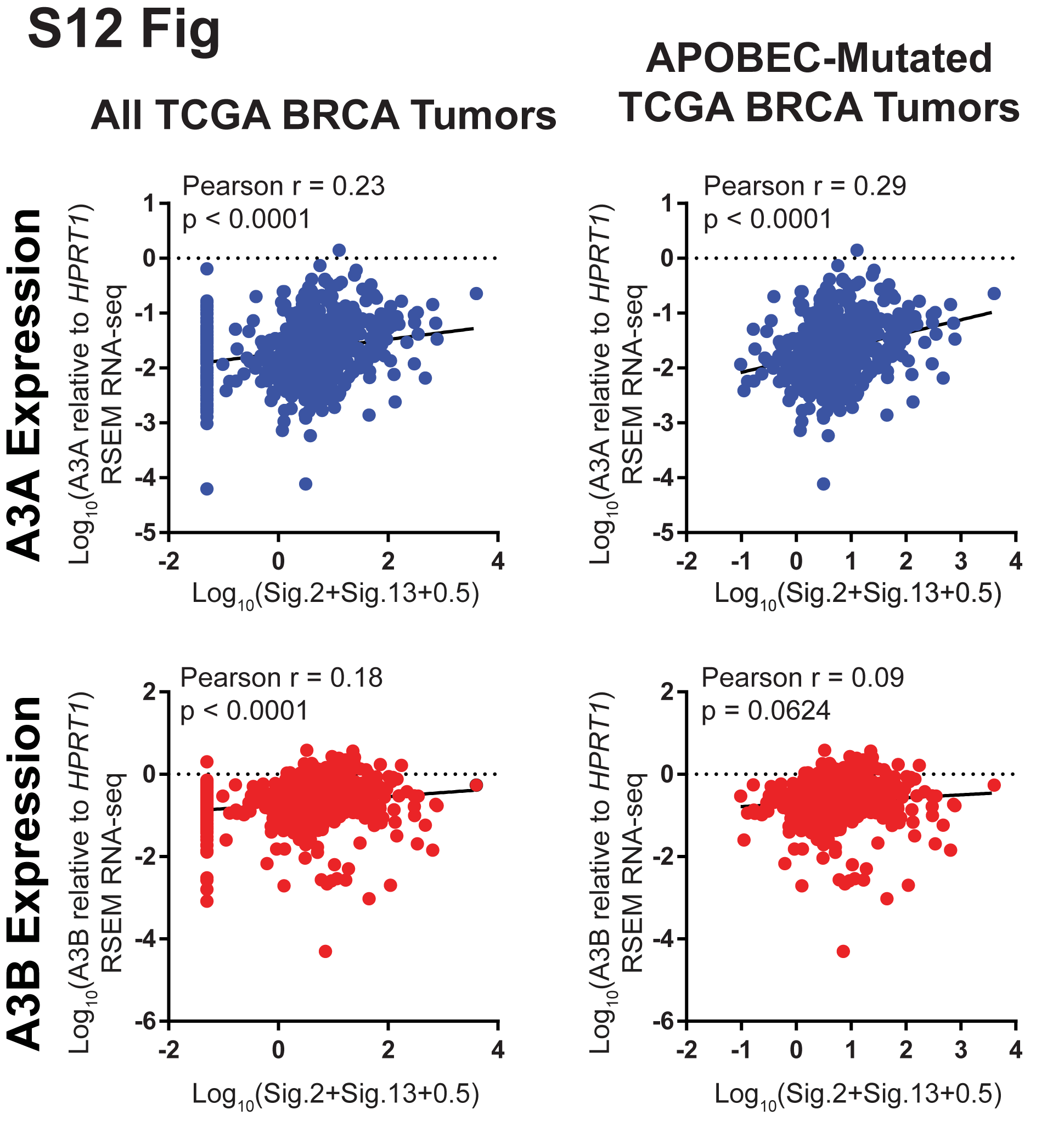

Supplement: S12 Fig — The mutations utilized in Fig 5 were deconvoluted into COSMIC mutation signatures. The number of mutations in Signatures 2 and 13 (indicative of APOBEC-induced mutation) were summed and compared to the A3A and A3B mRNA transcript levels for 577 primary BRCA tumors or 465 APOBEC-mutated BRCA tumors analyzed by the Mutational Patterns R package. (TIF) [file pgen.1008545.s013.tif]

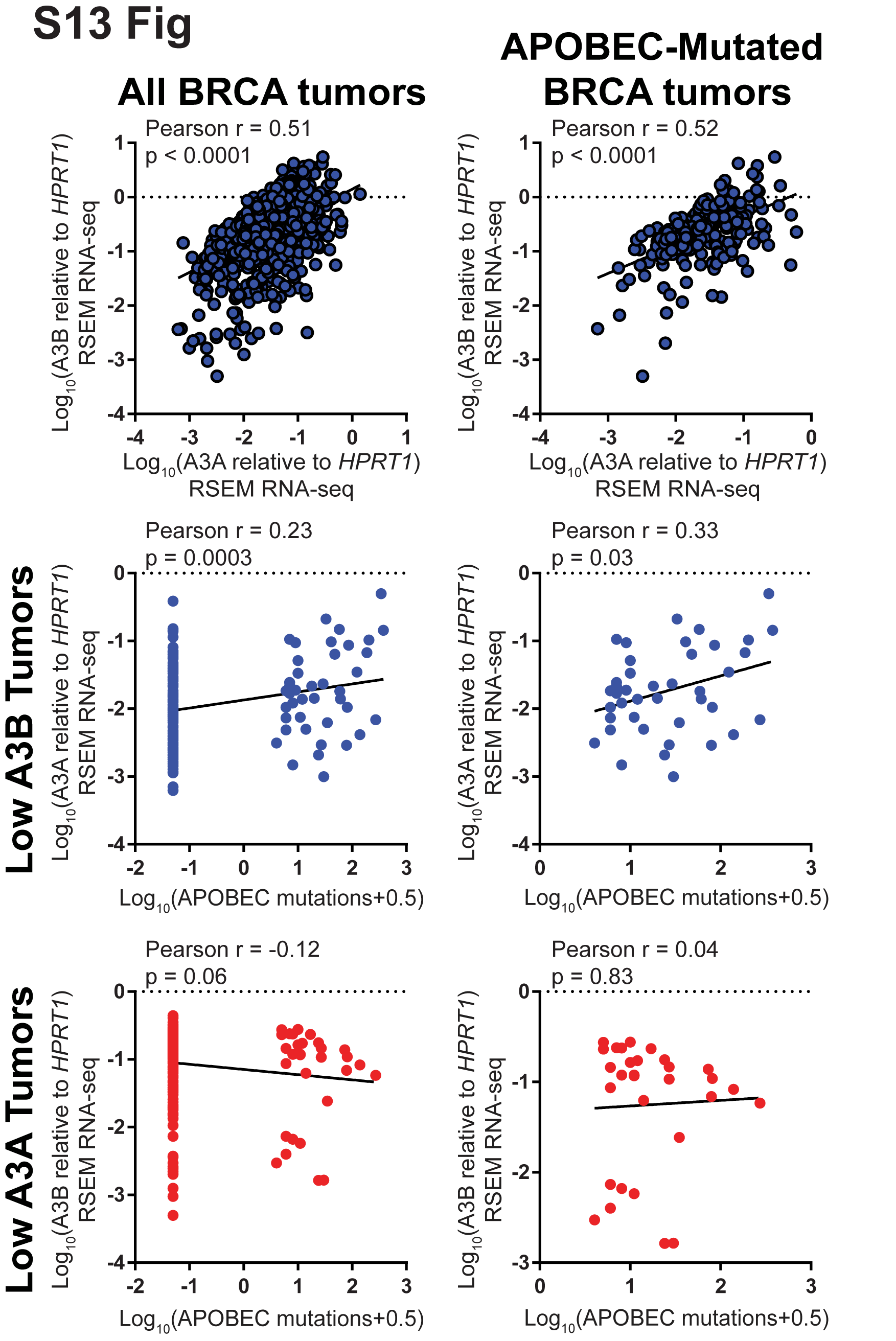

Supplement: S13 Fig — In primary BRCA tumors and the subset of APOBEC-mutated BRCA tumors, A3A expression positively correlates with A3B expression. However, in tumors restricted to the lowest quartile of A3B expression, the number of APOBEC-induced mutations still correlates with A3A mRNA transcript level. No correlation is observed between A3B expression and APOBEC mutagenesis in BRCA tumors expressing A3A at low levels. (TIF) [file pgen.1008545.s014.tif]

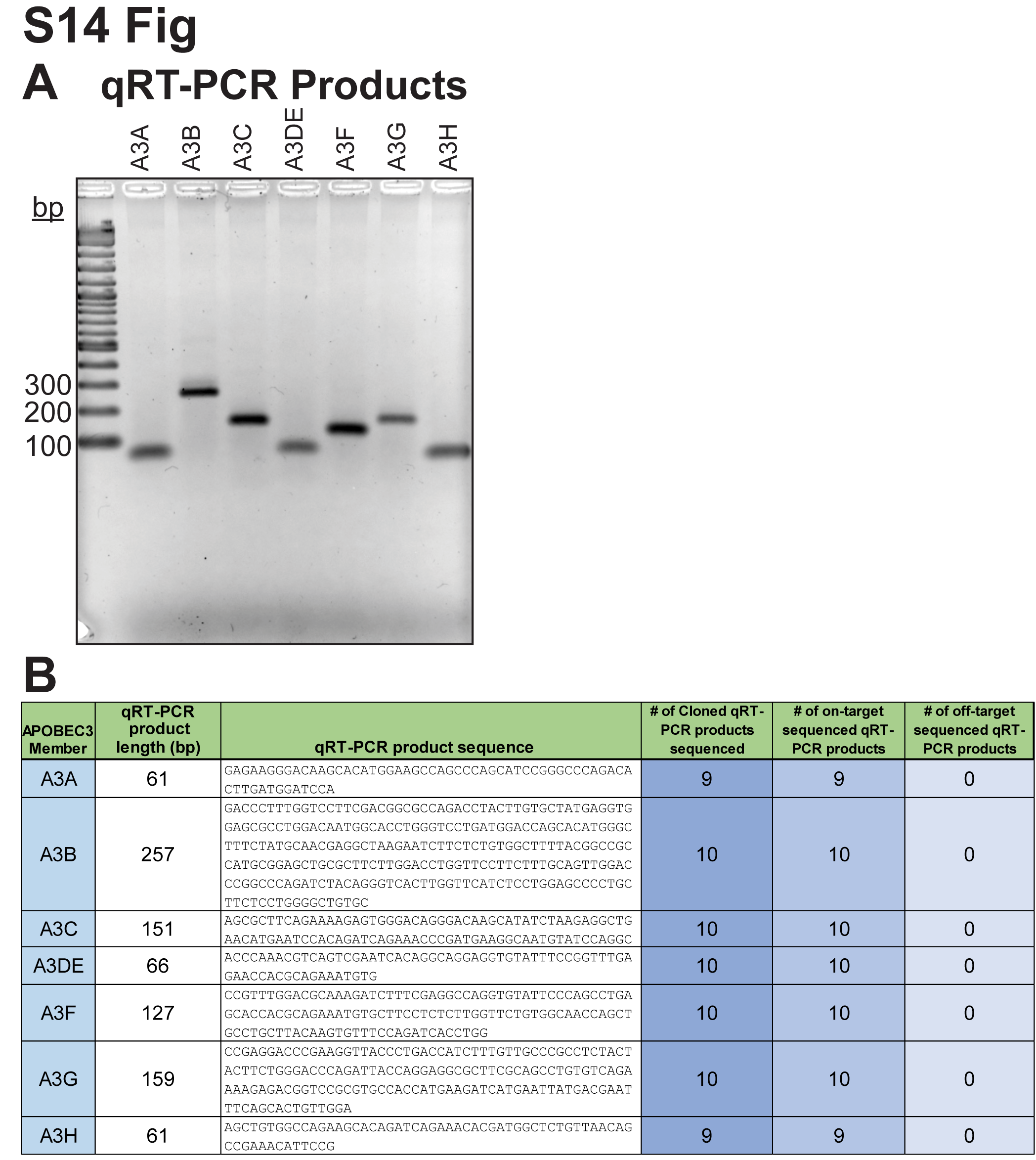

Supplement: S14 Fig — (A) Endpoint qRT-PCR products for measuring the expression of individual APOBEC3 family members were separated based on size by agarose gel electrophoresis. All reactions produce a single product of the expected size for specific APOBEC3 family member assessed. (B) qRT-PCR products were cloned into the pCR-Blunt DNA vector and 9–10 independent clones were sequenced to verify the products were on-target. All sequences aligned to the target amplification region. (TIF) [file pgen.1008545.s015.tif]
